# Strong Lensing by Galaxies

**DOI:** 10.1007/s11214-024-01105-x

**Published:** 2024-11-08

**Authors:** A. J. Shajib, G. Vernardos, T. E. Collett, V. Motta, D. Sluse, L. L. R. Williams, P. Saha, S. Birrer, C. Spiniello, T. Treu

**Affiliations:** 1 Department of Astronomy and Astrophysics, University of Chicago, Chicago, IL 60637 USA; 2 Kavli Institute for Cosmological Physics, University of Chicago, Chicago, IL 60637 USA; 3 Institute of Physics, Laboratory of Astrophysics, Ecole Polytechnique Fédérale de Lausanne (EPFL), Observatoire de Sauverny, 1290 Versoix, Switzerland; 4 Department of Astrophysics, American Museum of Natural History, Central Park West and 79th Street, New York, NY 10024-5192 USA; 5 Department of Physics and Astronomy, Lehman College of the CUNY, Bronx, NY 10468 USA; 6 Institute of Cosmology and Gravitation, University of Portsmouth, Burnaby Rd, Portsmouth, PO1 3FX UK; 7 Instituto de Fisica y Astronomia, Facultad de Ciencias, Universidad de Valparaiso, Avda. Gran Bretana 1111, Valparaiso, Chile; 8 STAR Institute, Quartier Agora - Allée du six Aout, 19c B-4000 Liége, Belgium; 9 School of Physics and Astronomy, University of Minnesota, 116 Church Street SE, Minneapolis, MN 55455 USA; 10 Physik-Institut, University of Zurich, Winterthurerstrasse 190, 8057 Zurich, Switzerland; 11 Kavli Institute for Particle Astrophysics and Cosmology and Department of Physics, Stanford University, Stanford, CA 94305 USA; 12 SLAC National Accelerator Laboratory, Menlo Park, CA 94025 USA; 13 Department of Physics and Astronomy, Stony Brook University, Stony Brook, NY 11794 USA; 14 Department of Physics, University of Oxford, Denys Wilkinson Building, Keble Road, Oxford, OX1 3RH UK; 15 INAF – Osservatorio Astronomico di Capodimonte, Via Moiariello 16, 80131 Naples, Italy; 16 Department of Physics and Astronomy, University of California, Los Angeles, 430 Portola Plaza, Los Angeles, CA 90095 USA

**Keywords:** Gravitational lensing: strong, Galaxies: elliptical and lenticular, cD, Galaxies: structure, Galaxies: evolution, Cosmological parameters

## Abstract

Strong gravitational lensing at the galaxy scale is a valuable tool for various applications in astrophysics and cosmology. Some of the primary uses of galaxy-scale lensing are to study elliptical galaxies’ mass structure and evolution, constrain the stellar initial mass function, and measure cosmological parameters. Since the discovery of the first galaxy-scale lens in the 1980s, this field has made significant advancements in data quality and modeling techniques. In this review, we describe the most common methods for modeling lensing observables, especially imaging data, as they are the most accessible and informative source of lensing observables. We then summarize the primary findings from the literature on the astrophysical and cosmological applications of galaxy-scale lenses. We also discuss the current limitations of the data and methodologies and provide an outlook on the expected improvements in both areas in the near future.

## Introduction

This review article discusses applications of galaxy-scale strong lenses to study the properties of the deflector galaxies, that is, the central lensing galaxies. These have so far typically been massive elliptical galaxies at $0.1 \lesssim z \lesssim1$ and strong lensing has been chiefly applied to study their internal structure and composition. However, a large sample of strong lenses with deflectors other than massive ellipticals is expected to be discovered in this decade from the upcoming deep sky surveys. We can also gain important insights into the formation and evolution of the deflectors by comparing their structural properties (e.g., the logarithmic slope of the density profile and the dark matter fraction) across cosmic times. Furthermore, in this review article, we present cosmological applications of the galaxy-scale lenses that do not require time delay information – that is, measuring cosmological parameters such as the matter density parameter $\Omega_{\mathrm{m}}$ and the dark energy equation-of-state parameter $w_{\mathrm{de}}$. Cosmological application involving the time delay measurements, that is, measuring primarily the Hubble constant, is reviewed by Birrer et al. (2024). Additionally, Vegetti et al. (2023) review the application of galaxy-scale lensing to study sub-galactic structures of dark matter.

In this introductory section, we provide a brief description of the lensing phenomenology (Sect. 1.1) and discuss the advantages of strong-lensing observables in comparison with other probes of galaxy mass, such as stellar dynamics (Sect. 1.2). The remainder of this review article is organized as follows. In Sect. 2, we highlight the significant historical results involving galaxy-scale lenses and introduce several prominent lens samples. In Sect. 3, we describe the strong-lensing observables and their modeling and analysis methods. We discuss the application of galaxy-scale strong lensing to study galaxy properties and evolution in Sect. 4 and to constrain cosmological parameters in Sect. 5. Next, in Sect. 6, we discuss open issues – both in technical aspects and scientific questions – and provide future outlooks. We conclude the review article in Sect. 7.

### Brief Description of Lensing Phenomenology at the Galaxy-Scale

**Fig. 1:**
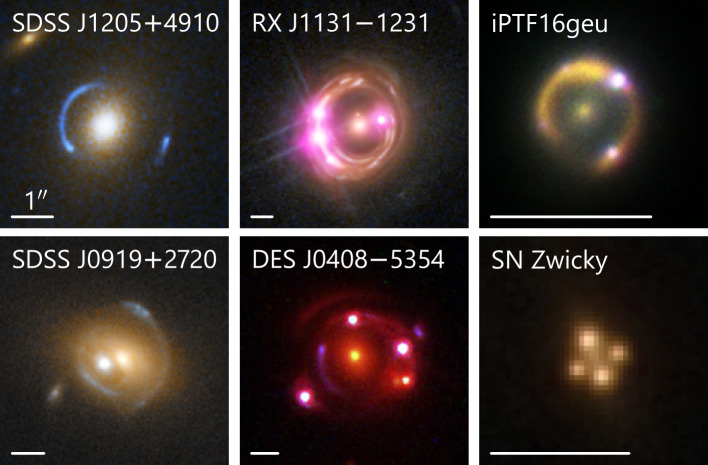
Examples of galaxy-scale lenses with different types of background sources. These are false color images created from multi-band *HST* imaging, and in some cases, also combined with the *Chandra* X-ray data (RX J1131−1231) and the Keck Observatory IR imaging (iPTF16geu). The first column shows two lenses with background galaxies without any resolved point source (Bolton et al. 2006; Courbin et al. 2012). The second column shows two lensed quasar systems (Suyu et al. 2013; Shajib et al. 2020). The third column shows two lensed supernovae (Goobar et al. 2017; Pierel et al. 2023). The white bar in each panel represents . *Image credits: NASA, ESA, A. Bolton, the SLACS team,* Chandra*, A. J. Shajib, W. M. Keck Observatory, T. Li, and J. Pierel*

The background extended source in a galaxy-scale strong lens can be lensed into multiple arcs or a complete Einstein ring. Multiple point images will also appear if there is a point source within the background galaxy, for example, an active galactic nucleus (AGN) or quasar, or a supernova (see Fig. 1). The former type of system is called galaxy–galaxy lenses. In contrast, the latter is usually referred to as ‘quads’ (for the case of four detected point images) or ‘doubles’ (for the case of two detected point images). The different manifestations of strong lenses – the appearance of arcs or a full Einstein ring, or the number of point images – depend on the position of the source with respect to the lens caustics (as introduced in Saha et al. 2024). Strong lensing of point sources can provide three types of observables: image positions, image magnification ratios, and time delays between the images. It follows from the lensing theory that all three are properties of the Fermat potential or the arrival-time surface. Images form at the local extrema – minima, saddle points, and maxima – of this potential. Magnification is inversely proportional to the determinant of the Fermat potential’s Jacobian matrix. Lastly, time delays are the differences in the Fermat potential at the image locations (for a detailed explanation, see Saha et al. 2024).

However, not all of these observables are available for every lens. Image positions are almost always observable for point-source lens systems. Image flux ratios, while also always observable, can only be turned into actual magnifications when the intrinsic source flux is known. However, interpreting these magnifications during modeling requires extra care, as they can be affected by more complex features in the galaxy-scale mass distribution, for example, baryonic disks (Hsueh et al. 2016, 2017), microlensing by individual stars and planets in the lensing galaxy (see Vernardos et al. 2024), intermediate-mass-scale structures like dark matter subhalos (see Vegetti et al. 2023), or dust extinction by the lens galaxy (e.g., Motta et al. 2002; Mediavilla et al. 2005). Time delays can be obtained only for variable point sources, like quasars, supernovae, and, in the future, gravitational waves and fast radio bursts (see Birrer et al. 2024). Measuring time delays can be a difficult task, especially for quasars, which require long-term monitoring spanning from a few seasons to years (e.g., Eigenbrod et al. 2005; Bonvin et al. 2016; Millon et al. 2020).

The above description of point-source lensing must be slightly modified for extended sources comparable in size to the lens caustics. Instead of point-like image positions, the light from such a source is spread into an extended area in the image plane and then further smeared by the point spread function. As a result, it is hard to know *a priori* how the observed flux of the multiple images of the source on the lens plane traces back to the same location on the source plane. Therefore, even if the actual source brightness is known, a lens model is required to compute the magnification field (i.e., the Jacobian of the lens potential) across the lens plane. Due to their size, extended sources are not variable on time scales relevant for lensing. Thus, time delays are not observable without a variable point source. Note that extended arcs from the point source’s host galaxy are also usually present for a lens system that includes a point source. Therefore, we can constrain the lens model by simultaneously utilizing the lensed arcs’ flux distribution and the point-image positions.

### Unique Advantages of Lensing as a Probe of Galaxy Structure

Other than lensing, the only commonly used probe of the mass distribution in galaxies is kinematics, that is, the velocity dispersion or streaming motions of stars, gas, or globular clusters. For this to be fully informative of the mass distribution, however, spatially resolved measurements are necessary (Cappellari 2016), which is often limited to nearby galaxies at $z \le0.5$ due to the sizeable observational cost, or due to the smaller size on the sky of objects at higher redshifts. Using individual stars’ motions, for example, from *Gaia*, to map a galaxy’s mass is only limited to our Milky Way (e.g., Nitschai et al. 2020). Thus, an aperture-integrated velocity dispersion measurement (from either long-slit or integral field spectroscopy) is the only possible dynamical observable for galaxies other than ours. However, strong-lensing observables are obtained from high-resolution imaging data, which are much more informative than the dynamical observables for galaxies beyond the local Universe ($z \gtrsim 0.03$, e.g., Smith et al. 2015). This is especially advantageous for studying the lens galaxies since the lensing signal does not depend on the surface brightness of the galaxy being studied, unlike stellar kinematics, but on the combination of the lens galaxy mass and the source brightness. Strong lensing can provide ∼1–2% constraints on the mass enclosed within the Einstein radius from imaging data alone. To obtain a similarly precise mass constraint from dynamical observations at high redshift, either integration times longer by a factor $\mathcal{O}(10)$ are needed on current facilities, or we must wait for better quality adaptive optics systems planned for future extremely large telescopes.

Furthermore, the dynamical observables have their own intrinsic degeneracies, that is, the mass–anisotropy degeneracy (e.g., Treu and Koopmans 2002). There is an important complementarity between lensing and dynamical observables, which can be used to break their corresponding degeneracies (e.g., Courteau et al. 2014). Finally, gravitational lensing responds to both baryonic and dark matter without any assumptions on their dynamical state, regardless of whether it is in equilibrium or not.

## Historical Background

This section provides a historical note on the initial discoveries of galaxy-scale strong lenses (Sect. 2.1). Then, in Sect. 2.2, we briefly introduce several prominent samples of galaxy-scale lenses that have contributed to the major science applications described in Sect. 4 and Sect. 5. Additional references can be found in the review by Treu (2010).

### Initial Discoveries of Strong Lensing Systems

The first strong lens, the double quasar 0957+561A, B, was discovered in 1979 by Walsh et al. (1979). The system consists of two images of the quasar separated by . The authors first offered a ‘conventional’ interpretation that the two images are different, individual quasars that happen to be close to each other and share the same physical characteristics. Since no gravitational lens was known before that, their less conventional view was that the two are multiple images of the same source. Subsequent work showed that the lensing hypothesis was correct. For example, the structure of the radio jets emanating from the two images of the quasar is consistent with them being mirror imaged, as one would expect for a minimum and a saddle-point image (Gorenstein et al. 1988; Garrett et al. 1994). The discovery of the first quadruply imaged quasar, PG 1115+080, was announced the following year (Weymann et al. 1980). It was initially called a ‘triple’ because the second arriving minimum and its neighboring saddle point were too close to be resolved. These two lenses’ discoveries opened up a new field in astrophysics: observations of multiply-imaged, ‘strong’ gravitational lenses. Discoveries of other types of lensed sources followed: for example, the first dust-obscured Seyfert 2 AGN, IRAS F10214+4724, was detected in the infrared (IR), and later identified as being lensed (Eisenhardt et al. 1996; Lehar and Broadhurst 1996).

The increasing number of detections of such lensed systems spurred a lens modeling effort. The initial studies that used more detailed models beyond a point-like lens mass appeared in the early 1980s (Young et al. 1980, 1981b,a), and already recognized “that there are several plausible ways to reproduce the observations”, foreshadowing the importance of lens model degeneracies. The role of the lens mass granularity due to individual stars in the lensing galaxy was also recognized very early on (Chang and Refsdal 1979; Young 1981) and later grew into the rich sub-field of extragalactic stellar microlensing (see Vernardos et al. 2024 for a review).

### Prominent Samples of Galaxy-Scale Lenses

In this subsection, we briefly introduce some of the most prominent samples of galaxy-scale lenses that had an impact on the science applications presented in Sect. 4 and Sect. 5. Note that this is not a complete list of all the discovered galaxy-scale lenses. The specifics of lens searching and discovery to build samples like these are reviewed by Lemon et al. (2024), which we refer the reader to for recent developments in the search methods and discoveries.

#### MG-VLA Survey-Based Samples

The first systematic search for strongly lensed systems at radio wavelengths took place in the eighties within the MIT–Greenbank–Very Large Array (MG-VLA) survey (Lawrence et al. 1986). This survey discovered a few famous radio-loud lensed quasars among thousands of radio sources scrutinized with high resolution by the Very Large Array (VLA). The Jodrell Bank–VLA Astromtric Survey (JVAS; Patnaik et al. 1992; King et al. 1996), and its successor, the Cosmic Lens All Sky Survey (CLASS; Myers et al. 1995) was the largest survey carried out for a long time. This survey targeted the whole northern sky ($0^{\circ} < \textrm{Dec} < 75^{\circ}$) for multiple images (separated by ) among flat spectrum radio sources brighter than 30 mJy. CLASS discovered 22 new systems, among which twelve are doubles, nine are quads, and one displays six images (Browne et al. 2003).

#### The CASTLES Sample

The CfA-Arizona Space Telescope LEns Survey (CASTLES^1^) is a follow-up *Hubble Space Telescope* (*HST*) imaging survey of ∼100 galaxy-scale lenses^2^ known at the time, some from previous surveys such as CLASS (Muñoz et al. 1998) and others from serendipity or targeted surveys (for details, see Lemon et al. 2024). This survey collected the first uniform ensemble of high-resolution images of known galaxy-scale lens systems, including both galaxy–quasar and galaxy–galaxy lenses. An account of early systematic lens searches, which generally unveiled small samples of less than six systems, can be found in Claeskens and Surdej (2002).

#### Samples of Lensed Sub-Mm Galaxies

Lens searches in the sub-mm have proved to be efficient in finding hundreds of lensed dusty star-forming galaxies at high redshift ($z \sim1$–4) with high purity in the candidate sample, using the sharp cutoff in the luminosity function for these galaxies (see Lemon et al. 2024). The initial samples detected using this technique came from the *Herschel* Astrophysical Terahertz Large Area Survey (HATLAS) and the *Herschel* Multi-tiered Extragalactic Survey (HerMES; Negrello et al. 2010, 2017). Since these samples of lenses are source-selected, the selection function of the lens galaxies is less affected than for lens-selected samples, thus providing an advantageous avenue to study galaxy properties (e.g., Dye et al. 2014, 2018; Amvrosiadis et al. 2018; Maresca et al. 2022).

#### SDSS-Based Samples

The Sloan Lens ACS (SLACS) survey discovered 85 galaxy–galaxy lenses from the Sloan Digital Sky Survey (SDSS) spectroscopic data by identifying multiple redshifts in the fiber (with 3 diameter) spectra. The SLACS survey also followed these systems up with multi-band *HST* imaging (Bolton et al. 2006; Auger et al. 2009). The sample was expanded with 40 new systems with smaller deflector masses by the SLACS for the Masses (S4TM) sample (Shu et al. 2015). In addition to galaxy–galaxy lenses, the SDSS Quasar Lens Search (SQLS) discovered a sample of 28 galaxy–quasar lenses using SDSS multicolor imaging data (Oguri et al. 2006). More galaxy–quasar lens systems were discovered from joint SDSS and UKIRT Infrared Deep Sky Survey (UKIDSS) data by the Major UKIDSS–SDSS Cosmic Lens Survey (MUSCLES; Jackson et al. 2012).

#### CFHTLS-Based Samples

The Strong Lensing Legacy Survey (SL2S; Gavazzi et al. 2012) discovered a sample of ∼35 galaxy–galaxy lenses from the Canada–France–Hawaii Telescope Legacy Survey (CFHTLS) data. Some newer lens samples have also been discovered from this survey (More et al. 2016a; Paraficz et al. 2016).

#### BOSS-Based Samples

The Baryon Oscillation Spectroscopic Survey Emission-Line Lens Survey (BELLS) discovered ∼30 galaxy–galaxy systems from the Baryon Oscillation Spectroscopic Survey (BOSS) and obtained *HST* imaging for them (Brownstein et al. 2012). This survey was later expanded into the BELLS for the GALaxy-Ly$\alpha$ EmitteR sYstems (BELLS GALLERY) survey, where the source galaxies are specifically Ly$\alpha$ emitters (Shu et al. 2016). A sample of 13 strongly lensed quasars has also been discovered from the BOSS data (More et al. 2016b).

#### Other Samples and Ongoing Efforts from Recent Surveys

Numerous large-area sky surveys have recently discovered several other lens samples. The STRong-Lensing Insights into the Dark Energy Survey (STRIDES) collaboration has discovered ∼30 quadruply lensed quasar systems from the Dark Energy Survey (DES) data – often in combination with data from other sky surveys – and obtained multi-band *HST* imaging of them in IR, optical, and ultra-violet (UV) bands (Shajib et al. 2019; Schmidt et al. 2023). Many galaxy–galaxy lens candidates have also been identified in the DES data (Jacobs et al. 2019b,a; Rojas et al. 2021; Tran et al. 2022). In addition to the DES, surveys such as the Canada–France Imaging Survey (CFIS), *Gaia*, the Hyper Suprime-Cam (HSC) survey, the Kilo Degree Survey (KiDS), and the Panoramic Survey Telescope and Rapid Response System (Pan-STARRS) have provided a plethora of newly discovered galaxy-scale strong lenses (e.g., Petrillo et al. 2017, 2019; Agnello et al. 2018; Krone-Martins et al. 2018; Delchambre et al. 2018; Lemon et al. 2018, 2020; Cañameras et al. 2021; Savary et al. 2022; Li et al. 2021; Lemon et al. 2022; Wong et al. 2022). Most of these samples still contain candidate lenses and require spectroscopic confirmation (by measuring the redshifts) and high-resolution imaging (to perform lens modeling) for the science applications described in Sect. 4 and Sect. 5. See Lemon et al. (2024) for a detailed discussion of the recent and ongoing efforts.

## Observables and Analysis Methods

This section describes the strong-lensing observables (Sect. 3.1) and the analysis techniques to constrain galaxy properties from them. We describe the lens modeling methods in Sect. 3.2 and the commonly used models in Sect. 3.3. We outline the Bayesian hierarchical framework in Sect. 3.4, which allows inferring population characteristics of galaxies from a sample of lenses. Lastly, non-lensing observables most commonly combined with strong lensing ones are presented in Sect. 3.5.

### Lensing Observables

The two types of lensing observables for galaxy-scale lenses are imaging of the lensing system (Sect. 3.1.1) and the time delay between a pair of images from a point source (Sect. 3.1.2).

#### Imaging of the Lens System

The most common and informative lensing observables result from imaging data with angular resolution much better than the Einstein radius. An extended source can be lensed into clearly identifiable arcs that can form partial or complete Einstein rings (see Fig. 1). The conjugate points on the arcs, that is, locations that are traced back to the same location in the source plane, can be used to simultaneously constrain a lens model (through its deflection angles and magnification) and reconstruct the surface brightness of the source that is *a priori* unknown. Although simple models can be constrained from any image that displays lensing features with sufficient signal-to-noise ratio, high-resolution imaging from space- or ground-based telescopes offers many more observational constraints (i.e., conjugate pixels). This is crucial for exploring more sophisticated models, which are required for precise science applications (e.g., see Sect. 4 and Sect. 5).

If the background source contains a point-like emitting region – for example, a quasar or a supernova – the positions of its multiple images (i.e., conjugate points) can be extracted from the data and used as constraints for a lens model. Although the simple addition of two or four conjugate points may initially seem insignificant compared to the hundreds of pixels that correspond to the extended source, the flux contained in the corresponding pixels may be brighter than the entire host galaxy, for example, in the case of an AGN, and their positions can be constrained with sub-pixel accuracy (e.g., Shajib et al. 2019; Schmidt et al. 2023). Hence, these conjugate points have a strong effect on the resulting model and must be treated separately. In addition to the astrometry of the point-like images, their magnification ratio (or flux ratio) can be used as an observational constraint. However, image magnifications are susceptible to micro- and milli-lensing, dust extinction,^3^ and the effect of higher order moments in the mass distribution of the lens usually attributed to the complex, non-linear physics of baryons, for example, galactic disks and bars (Hsueh et al. 2016, 2017). Therefore, many lensing analyses involving systems with such point-like sources choose not to use magnification ratios as constraints (e.g., Shajib et al. 2019). However, suppose the microlensing and dust extinction effects can be incorporated and quantified within a lens model. In that case, any residual flux ratio anomaly would signal a departure from the smooth macro-model for the deflector galaxy and thus could indicate the presence of sub-galactic dark matter structures within the galaxy-scale halo (e.g., Mao and Schneider 1998; Nierenberg et al. 2017; Gilman et al. 2020). Such detection of dark substructures can provide important insights into the nature of the dark matter, as described in Vegetti et al. (2023).

Lens modeling by simultaneously fitting the imaging data in multiple bands (from radio wavelengths to UV) has become commonly employed in the literature when such data are available (e.g., Dye et al. 2014; Oldham and Auger 2018; Shajib et al. 2019; Young et al. 2022; Tan et al. 2024). Such multi-band modeling has the advantage of adding constraints in regions of the images that may be poorly detected in some wavelength ranges and deblending the lensing galaxy from the images and arcs. The main drawback is the increase in model complexity due to the wavelength dependence of the lens galaxy and source morphology. However, such multi-band lens modeling is expected to be ubiquitous in the upcoming decade, with multi-band data being more available thanks to the current and upcoming facilities such as the *JWST* and the Vera Rubin Observatory (Shajib et al. 2024). Several automated modeling pipelines (for both single-band and multi-band data) are being developed to tackle the computational aspect of modeling very large lens samples (Oguri and Marshall 2010; Collett 2015) to be discovered by the Rubin Observatory, *Euclid*, and the *Roman Space Telescope* (e.g., Chan et al. 2015; Nightingale et al. 2018; Shajib et al. 2021; Etherington et al. 2022; Schmidt et al. 2023; Tan et al. 2024).

#### Time Delay

If the background source is a variable point source, for example, a quasar or a supernova, the delay between the arrival times of photons at its multiple different images can be measured through long-term monitoring that spans from a few months to decades (e.g., Eigenbrod et al. 2005; Bonvin et al. 2016; Millon et al. 2020). The time delays are variant under the well-known mass-sheet degeneracy (MSD) in lensing, unlike the imaging observables (Falco et al. 1985; Schneider and Sluse 2014). Thus, they can be combined with the imaging observables to break the MSD when constraining the potential of the lensing galaxy. However, such a combination of these observables requires a fiducial cosmological model since the time delays depend on the cosmology, particularly the Hubble constant. A more detailed discussion on the measurement of time delays is provided in Birrer et al. (2024).

### Lens Modeling Methods

Lens modeling is the process of constraining properties of the lens galaxy and the source from the lensing observables. Traditional methods are based on reconstructing the source light and lens potential to fit the data under some assumptions, such as regularization, and are described in Sect. 3.2.1. More recently, machine learning methods are being developed for these purposes, which we present in Sect. 3.2.2. It is beyond the scope of this review to provide a beginner’s guide to modeling galaxy-scale lenses, but we refer the reader to Saha et al. (2024) for an introduction to lens modeling. Readers interested in the science applications without needing a technical discussion on lens modeling and analysis techniques may go directly to Sect. 4 and refer back to the rest of this Sect. 3 as needed.

#### Likelihood-Based Inference

Such methods require a likelihood function that leads to an optimized model that can reproduce an observed multiply-imaged system down to the noise level. We note that whereas an optimized forward model aims to reproduce the data to the noise level, it is often difficult to achieve that in practice, often owing to the simplifying assumptions made in the model. As a result, fine-tuning the model complexity is commonly required through trial-and-error to meet the accuracy requirement for a given science case (e.g., Shajib et al. 2019; Schmidt et al. 2023). In general, the lens model consists of three main components: the background source’s flux distribution, the mass distribution in the lensing galaxy (or galaxies), and the flux distribution in the lensing galaxy (or galaxies). The likelihood function that measures the goodness-of-fit of the model to the data can be defined as

1 $$ \mathcal{L}(\boldsymbol{d} \mid\boldsymbol{m}) \propto\exp\left[ - \frac{1}{2}\left(\boldsymbol{d} - \boldsymbol{m}\right)^{T} {\Sigma}_{\boldsymbol{d}}^{-1}\left(\boldsymbol{d} - \boldsymbol{m}\right) \right], $$

where $\boldsymbol{d}$ is the vector of pixel values in the data and

2 $$ \boldsymbol{m} \equiv\boldsymbol{m}(\boldsymbol{\xi}_{\mathrm{mass}}, \ \boldsymbol{\xi}_{\mathrm{source}},\ \boldsymbol{\xi}_{ \mathrm{light}}) $$

is the model-computed flux in the data pixels. Here, $\boldsymbol{\xi}_{\mathrm{mass}}$ is the set of model parameters defining the lens mass distribution, $\boldsymbol{\xi}_{\mathrm{source}}$ is the set of model parameters defining the source’s flux distribution, $\boldsymbol{\xi}_{\mathrm{light}}$ is the set of model parameters defining the lens galaxy’s flux distribution, and ${\Sigma}_{\boldsymbol{d}}$ is the covariance matrix of the data. Some studies choose to subtract the lens galaxy’s flux distribution from the imaging data before lens modeling (e.g., Bolton et al. 2008b). Given that the lens flux is loosely related to the lensing phenomenon only through mass-follows-light arguments that are not strictly required, we omit it in the following discussion for brevity. This likelihood function can be extended in a Bayesian framework to include prior (or regularization) terms on the source (Warren and Dye 2003; Treu and Koopmans 2004; Koopmans 2005), or used to compute the Bayesian evidence (Suyu et al. 2006; Shajib et al. 2020; Vernardos and Koopmans 2022) and perform model comparisons.

When the lensed arcs from an extended source are resolved, each pixel is a constraint for the lens model. Given a deflection field $\boldsymbol{\alpha}(\boldsymbol{\theta})$, which depends on the lens mass distribution through $\boldsymbol{\xi}_{\mathrm{mass}}$, the lens equation

3 $$ \boldsymbol{\beta}(\boldsymbol{\theta}) = \boldsymbol{\theta}- \boldsymbol{\alpha}(\boldsymbol{\theta}) $$

can be used to map any position $\boldsymbol{\theta}$ on the image plane to the corresponding position $\boldsymbol{\beta}$ on the source plane (for a detailed explanation of the strong lensing formalism, see Schneider et al. 1992, Meneghetti 2021, or Saha et al. 2024). We can then easily compute the lensed flux at any location on the image plane as

4 $$ I(\boldsymbol{\theta}) = S[\boldsymbol{\beta}(\boldsymbol{\theta})], $$

where $S$ is the light distribution of the source that depends on $\boldsymbol{\xi}_{\mathrm{source}}$, and we use the fact that lensing conserves surface brightness. The dependence of $I(\boldsymbol{\theta})$ on the mass through $\boldsymbol{\beta}(\boldsymbol{\theta})$ is almost always non-linear, which means that we cannot directly (i.e., through a linear inversion) solve Eq. (3) to obtain the true parameters $\boldsymbol{\xi}_{\mathrm{mass}}$, even when $S$ (and equivalently the values of $\xi_{\mathrm{source}}$) is perfectly known.

In practice, $S$ is an unknown that must be solved simultaneously with the lens potential and requires special attention. The most straightforward choice for it is an analytic function, for example, a Sérsic profile (Sérsic 1968), whose parameters are treated in the same forward-modeling way as for the lens potential. However, several more advanced techniques have been developed over the years that allow a free-form reconstruction of the source, each with its advantages and disadvantages. The semi-linear inversion technique of Warren and Dye (2003) uses a regular grid of pixels to approximate the source brightness. Although the degrees of freedom are much higher in this case, the use of regularization or prior terms of a specific form can greatly facilitate obtaining the best-fit solution. If a prior is used with quadrature terms of the source pixel brightness values, then the derivative of the posterior probability function^4^ can be obtained analytically given $\boldsymbol{\xi}_{\mathrm{mass}}$. As a result, the source reconstruction turns into a linear inversion problem for a given set of non-linear parameters within $\{\boldsymbol{\xi}_{\mathrm{mass}}, \boldsymbol{\xi}_{\mathrm {source}}\}$, where $\boldsymbol{\xi}_{\mathrm{source}}$ now refers only to non-linear parameters of the source that are not solved through the linear inversion.

**Fig. 2:**
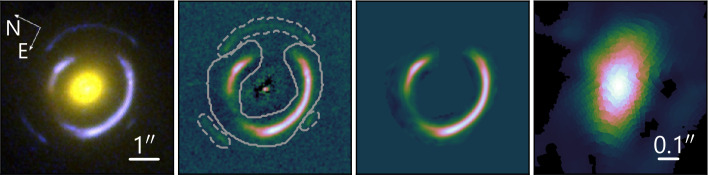
*First panel:* False-color image of the system SDSS J0946+1006 combining three *HST* filters (Sonnenfeld et al. 2012). *Second panel:* *HST* image in the F814W filter of the system with the galaxy light subtracted. This system has lensed arcs from multiple source galaxies at different redshifts, which are grouped with solid and dashed contours. *Third panel:* Model of the lensed arc from only the brightest source. *Fourth panel:* Corresponding source reconstructed on an adaptive grid using every data pixel and curvature regularization (for the analysis of the data, see Chap. 4 of Bayer 2021 and for the modeling method, see Vernardos and Koopmans 2022)

Typical choices of source regularization are gradient and curvature that impose smoothness on the source solution through its derivatives (a standard approach in this kind of problem, e.g., Press et al. 1992). Alternatively, Vegetti et al. (2014) and Nightingale et al. (2018) use adaptive regularization, which changes the degree of smoothing based on the source brightness, and both studies discuss that this scheme is vital for reconstructing compact sources. Other choices include a covariance kernel prior, which is based on observations of the galaxy brightness power spectrum and thus a more physically justifiable choice (Vernardos and Koopmans 2022), or a multi-scale regularization through the use of sparsity constraints on a wavelet representation of the source (Galan et al. 2021). Choosing a different basis to represent the source can in itself significantly reduce the number of degrees of freedom while still having enough flexibility to represent complex light profiles across different scales (Birrer et al. 2015; Tagore and Jackson 2016). This can be similarly achieved by reconstructing the source on an irregular, adaptive grid that can have increased resolution in the most magnified areas of the source, that is, near the caustics (Vegetti and Koopmans 2009; Nightingale and Dye 2015; Vernardos and Koopmans 2022). Figure 2 illustrates an example of lens modeling based on high-resolution *HST* imaging of a lensing system with a bright, extended source component. The source is reconstructed on an adaptive grid using curvature regularization (Chap. 4 of Bayer 2021).

**Fig. 3:**
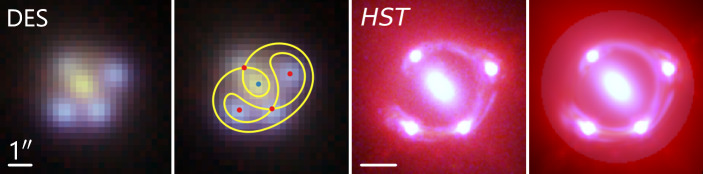
Lens modeling of a galaxy-scale lens system – here, the lensed quasar WGD J2038−4008. *First panel:* The false-color image of the system from the DES $giy$-bands, where only the point images are resolved (Agnello et al. 2018). *Second panel:* Illustration of a lens model based only on the quasar image positions (red points; Agnello et al. 2018). The blue point shows the center of the lensing galaxy, and the yellow lines trace the saddle-point contours on the arrival-time surface. *Third panel:* The false-color image of the system from 3-band *HST* imaging (in F160W, F814W, and F475X filters), where the lensed arcs from the quasar host galaxy can be seen in greater detail. *Fourth panel:* The pixel-level model of the *HST* imaging from Shajib et al. (2022). The white bars represent

If there is a point source within the extended source galaxy, then the positions of its point-like images provide additional constraints on the mass model as conjugate points. One approach to model the point-like source is to include the location of its multiple images as free parameters and then require the mass model to trace them back to the same location on the source plane through the lens equation. Alternatively, the point-source location can be free, and the predicted image positions must match the observed ones. In practice, the choice between the two approaches is based on computational and sub-grid effect arguments (see Keeton 2001b, for a more detailed presentation on the topic). Figure 3 illustrates an example of lens modeling based on high-resolution *HST* imaging of a lensing system with both a point-like and an extended source component, that is, a quasar and its host galaxy. In this case, the model for the extended host galaxy assumes an analytic Sérsic profile and an additional free-form component described by a basis set of shapelets (Birrer et al. 2015; Shajib et al. 2022).

#### Machine-Learning Based Parameter Extraction

Recently, machine learning (ML) based methods have been developed to extract lensing parameters – for example, the Einstein radius, power-law index, ellipticity, and shear parameters – from the imaging data (e.g., Hezaveh et al. 2017; Morningstar et al. 2019; Adam et al. 2022; Schuldt et al. 2023). Some studies also explored reconstructing the lens mass and source flux distributions using machine learning algorithms (e.g., Chianese et al. 2020; Karchev et al. 2022; Mishra-Sharma and Yang 2022; Biggio et al. 2023). In these approaches, a machine learning algorithm – usually a neural network – is trained using synthetic data since real examples of lens systems are not adequate in number for such data-intensive training. This leads to the critical caveat that the mass density profile in the simulated galaxies is based on empirical priors (e.g., Hezaveh et al. 2017) or cosmological simulations (e.g., Adam et al. 2022). Thus, the inferred parameters are prior-dependent – either empirical or physical – in a similar manner that the forward modeling approach depends on the adopted mass model and the associated priors.

Although ML-based inferences have yet to demonstrate their potential in science applications with real data, they are essential for quickly extracting lensing parameters with minimum human supervision. This is crucial for modeling huge samples of lenses like those expected from several large surveys upcoming in the 2020s (e.g., Park et al. 2021; Wagner-Carena et al. 2021). For such large datasets, the traditional forward modeling techniques would be unfeasible due to either computational or human time restrictions. Finally, ML-based inference provides a direct and fast way to constrain quantities of interest that can be otherwise too cumbersome to infer from the data through a traditional approach, for example, detecting individual or populations of dark subhalos or constraining the subhalo-mass function (Brehmer et al. 2019; Diaz Rivero and Dvorkin 2020; Coogan et al. 2020, 2022; Ostdiek et al. 2022a,b; Vernardos et al. 2020; Wagner-Carena et al. 2021; Zhang et al. 2022; Anau Montel et al. 2023). Note that this particular science application is discussed in detail in Vegetti et al. (2023).

### Lens Mass Models

Lens mass models can vary in complexity and eventually in the number of free parameters, reflecting that the mass distribution in galaxies is a non-trivial problem for which methodologies and algorithms are still evolving. Although it is possible to extract lensing information with free-form models and directly connect them to galaxy properties of interest (see Saha et al. 2024), lens modeling with simple parametrization has been the most common practice in the literature (e.g., Ritondale et al. 2019; Schmidt et al. 2023). We briefly introduce some commonly used models with simple parametrization in Sect. 3.3.1, discuss the degeneracies that impact them in Sect. 3.3.2, and describe free-form models in Sect. 3.3.3.

#### Simply Parametrized Mass Models

Keeton (2001a) provides a large catalog of simply parametrized models, which includes the commonly used ones such as the singular isothermal sphere or ellipsoid (SIS or SIE; Kormann et al. 1994), the pseudo-isothermal elliptical mass distribution (PIEMD; Kassiola and Kovner 1993), the softened power-law elliptical mass distribution (SPEMD; Barkana 1998), and the Navarro–Frenk–White (NFW; Navarro et al. 1996, 1997) profile. Defining the ellipticity in the potential makes lensing computation very efficient, even for complex radial profiles, since all the lensing quantities can be obtained either from the potential itself (e.g., for the time delay) or through numerical differentiation of the potential (e.g., for the deflection, convergence, or magnification; Kovner 1987; Golse and Kneib 2002). However, moderately elliptical potentials (e.g., with axis ratio $q \lesssim0.6$) can lead to unphysical shapes in the convergence (Kassiola and Kovner 1993) and also introduce implicit azimuthal variation or ellipticity gradient in the convergence (Gomer et al. 2023). For simply parametrized profiles with ellipticity defined in the convergence, computing deflection angle becomes computationally expensive without an analytical solution due to needing a 2D numerical integration. Among such elliptical convergence profiles, Tessore and Metcalf (2015) provide an analytical solution for the power-law radial form. Alternative parameterizations of the elliptical NFW convergence profile have also been devised (Oguri 2021; Heyrovský and Karamazov 2024). Shajib (2019) provides a computationally efficient general solution for any radial form using a superposition of elliptical Gaussian components (i.e., the multi-Gaussian expansion; Emsellem et al. 1994; van de Ven et al. 2010).

On top of such a simply parametrized profile describing the primary lens mass distribution, it is also often necessary to include a constant shear field (often shortened as XS) lensing potential to accurately model the distortions in the lensed arcs or the Einstein ring. These additional distortions can arise from nearby perturbers and large-scale structures (Keeton et al. 1997) or the additional angular structure in the central lensing galaxy or galaxies (Witt 1996; Hilbert et al. 2007; Gomer and Williams 2018, 2021; Barrera et al. 2021; Van de Vyvere et al. 2022a,b; Etherington et al. 2024). This constant shear field is commonly referred to as ‘external shear’ in the literature, pointing to the former of the two origins mentioned above. However, we recommend using the term ‘residual shear’ instead as a more general terminology. The magnitude of residual shear commonly exceeds 0.1, which is difficult to explain if this shear originates solely from the line-of-sight structures. Until the mid-2010s, the SIE+XS model has been the most popular choice to model large samples of galaxy-scale lenses (e.g., Bolton et al. 2008a; Sonnenfeld et al. 2013), adequate for the science application requirements at the time. Although simple SIE models can sufficiently constrain the Einstein radius, obtaining other important properties, such as the radial slope of the mass profile, requires models with additional degrees of freedom. Improved data quality and analysis techniques have allowed the use of such models (e.g., Ritondale et al. 2019; Shajib et al. 2021).

Simply parametrized lens models with larger degrees of freedom, for example, a superposition of a stellar component with a constant or varying mass-to-light ratio and a dark matter component usually described by the NFW profile, have been adopted by some studies (e.g., Treu et al. 2010; Sonnenfeld et al. 2015; Oldham and Auger 2018; Shajib et al. 2021). Although necessary for the addressed science questions, such mass profiles with more free parameters amplify the impact of degeneracies inherent to lensing. It is thus often necessary to constrain these additional parameters by incorporating non-strong-lensing observables such as stellar kinematics and weak lensing, or by incorporating informative priors (e.g., Sonnenfeld 2018; Shajib et al. 2021). We note that Sonnenfeld (2018) and Shajib et al. (2021) combined strong-lensing information with stellar kinematics or weak lensing using ‘summary observables’, such as the Einstein radius or the reduced slope $\xi_{\mathrm{rad}}$ (see Sect. 3.3.2 for definition), instead of simultaneously fitting the abovementioned complex model to the full lensing information in the imaging data.

While the additional degrees of freedom beyond the simple SIE model focus on the radial profile of the lens in most cases, azimuthal structures such as disky-ness, boxiness, ellipticity gradients, or isodensity twists may leave noticeable imprints in the lensed images. For point-like sources, the image flux ratios are the most susceptible to perturbations (Möller et al. 2003; Keeton et al. 2003, 2005). In contrast, for extended sources, the imprint is generally more subtle and detectable only from high-resolution imaging data (Van de Vyvere et al. 2022a,b). Fortunately, most of those structures arise from baryonic physics and may also be detectable in the luminosity profile of the lens (Van de Vyvere et al. 2022b).

#### Common Degeneracies in Simple Parametric Modeling

The degeneracies in lens modeling – both intrinsic in the data and stemming from the parametrization scheme – are discussed in Saha et al. (2024). Here, we summarize the common degeneracies that largely impact simply parametrized lens models for the convenience of the readers.

The MSD, which is intrinsic to imaging observables in lensing, originates from the mass-sheet transform (MST, Falco et al. 1985; Saha 2000)

5 $$ \kappa\to\kappa^{\prime} = \lambda\kappa+ 1 - \lambda, $$

where $\lambda$ is a constant. This equation implies a source position transformation (e.g., Schneider and Sluse 2014), where the unknown source position is altered as

6 $$ \beta\to\beta^{\prime} = \lambda\beta. $$

A more general but approximate degeneracy happens when $\lambda$ is not a constant anymore but depends on the position, that is, λ≡λ(θ) (Unruh et al. 2017; Wertz et al. 2018).

Most simply parametrized mass profiles artificially limit the MSD. For example, this is easily demonstrable for the power-law mass model, as the MST of a power law is mathematically not a power law anymore. Although MST-invariant quantities exist that the imaging observables can constrain (e.g., Wagner 2017; Wagner and Tessore 2018), commonly employed mass models are not parametrized based on those quantities. The standard practice in the literature to assume simply parametrized models, such as the power law, is usually validated on numerous non-lensing constraints demonstrating that the power-law model is a ‘good’ approximation for elliptical galaxy mass profiles (e.g., Thomas et al. 2007; Tortora et al. 2014; Bellstedt et al. 2018). The model-independent radial quantities constrained by the imaging data are the Einstein radius $\theta_{\mathrm{E}}$ and the MST-invariant ‘reduced slope’ defined as 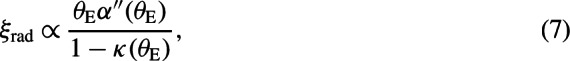
where  is the second derivative of the deflection angle (see Eq. 42 of Birrer 2021 for the full definition of $\xi_{\mathrm{rad}}$, also Kochanek 2021). For a power-law convergence profile $\kappa(\theta) \propto\theta^{-\gamma_{\mathrm{PL}} + 1}$, this quantity becomes $\xi_{\mathrm{rad}} = \gamma_{\mathrm{PL}} - 2$. As a result, the choice of a SIE+XS lens model fixes $\xi_{\mathrm{rad}} = 0$ and only extracts $\theta_{\mathrm{E}}$ from the imaging data.

Degeneracies in lens modeling can also arise from particular parametrizations of the lens model. One such example is the shear–ellipticity degeneracy, as the total shear can be redistributed between the ‘internal’ shear, arising from the ellipticity of the central deflector, and the external shear (Kassiola and Kovner 1993). Specifically, a SIE+XS model can be modeled with only an SIE model that has a quadrupole moment equaling 1/3^rd^ of the shear (An 2005). This degeneracy is particularly apparent when modeling with point-like image positions as the only constraints (Witt 1996).

#### Free-Form Models

In the most commonly used lens models, the main focus is on capturing the radial shape of the mass profile, while any azimuthal structure beyond an elliptical shape with residual shear is of secondary importance. Real galaxies can have more complicated mass profiles, with higher order moments present, such as disky-ness, boxiness, or bar- or disk-like components (e.g., Trotter et al. 2000; Claeskens et al. 2006; Hsueh et al. 2017; Frigo et al. 2019), radial dependence of the ellipticity or the orientation of the isodensity contours (i.e., ellipticity gradients, twists, or lopsidedness, e.g., Hao et al. 2006; Nightingale et al. 2019; Barrera et al. 2021), or even features that do not fit into a simple parametric description, such as merger products that are not yet completely relaxed and populations of substructures (e.g., satellite subhalos or perturbers along the line of sight). Detecting such deviations from the simple parametric profiles depends on numerous factors, such as their alignment with the smooth potential (Van de Vyvere et al. 2022b), the complexity of the brightness profile of the source (Vernardos and Koopmans 2022), the signal-to-noise ratio, etc. However, multipole components in the mass potential beyond the combined effect of ellipticity and residual shear have been recently detected in very-long-baseline interferometric observations of a strong lens (Powell et al. 2022). Although not accounting for such structures can bias the mass model by up to several percent – which is mostly acceptable except for time-delay cosmography (see Birrer et al. 2024) and for detecting dark matter substructure (see Vegetti et al. 2023) – their detection holds valuable information on the formation history and evolution of galaxies.

To this extent, free-form techniques have been developed that either entirely dismiss any parametric mass component and employ a grid of mass pixels to describe the lens potential (Saha and Williams 1997), or retain a parametric model as a first-order smooth component and combine it with a similar pixel grid that now focuses specifically on capturing higher order deviations (Koopmans 2005; Suyu et al. 2010). In both cases, regularization assumptions or priors on the free-form pixel grid are necessary to obtain a solution and to prevent the appearance of unphysical mass distributions. Existing techniques are based on forward models or extensions of the semi-linear inversion technique (Vernardos and Koopmans 2022).

The specific form of the regularization priors plays an important role in the quality of the obtained solutions. It has been shown that purely mathematically motivated priors (e.g., curvature) can lead to biased potentials as opposed to more physically driven ones, based on the observed light properties of real galaxies (e.g., Vernardos and Koopmans 2022). Galan et al. (2022) proposed a wavelet-based regularization technique that finds solutions that satisfy sparsity constraints. Biggio et al. (2023) completely replaced the pixel grid with a neural network. These approaches allow the use of purely data-driven regularization that is the most compatible with the data (without computing and comparing the Bayesian evidence). It remains to be seen how well these new and promising techniques can perform on real data and robustly recover deviations from smooth, parametric models that encode galaxy evolution.

One way to allow the prior to be less informative for free-form models is to marginalize over an ensemble of solutions. Such ensembles were first introduced for free-form models made up of mass tiles or pixels (Williams and Saha 2000; Saha and Williams 2004; Coles et al. 2014). In these and related works, the mass distribution is required to be non-negative and centrally concentrated in a broad sense. Within these prior conditions, models that correctly reproduce the observed positions of point-like image features are randomly sampled to form the ensemble of solutions. This ensemble of solutions is then effectively the posterior of the model parameters, which include all the mass pixel values, and the posteriors of model-predicted quantities can also be obtained from this ensemble (e.g., Williams and Saha 2000). The ensemble can be further filtered according to how well the whole image or the pixels on the lensed arcs from the extended source can be fitted (e.g., Denzel et al. 2021b).

Free-form models naturally allow a broad range of mass profile shapes, both radially and azimuthally, thus exploring the degenerate space of the mass profile shapes. The same effect can be obtained with simply parametrized models by combining posteriors from models with different parametric forms, albeit to the limited extent allowed by the variety of the adopted parametrizations (e.g., Suyu et al. 2014; Birrer et al. 2019; Shajib et al. 2022).

### Bayesian Hierarchical Framework

The Bayesian hierarchical framework can be used to constrain the population properties from a sample of strong lenses (e.g., Sonnenfeld et al. 2015). This framework also allows one to incorporate a selection function of the lensing galaxies and generalize the sample properties to the population of all galaxies that are of the same type as the lensing ones (e.g., Sonnenfeld et al. 2019). Within the hierarchical analysis, there are two levels of parameters: hyper-parameters that dictate the distribution of the parent population of the lens galaxies and parameters pertaining to individual lens galaxies sampled from the parent population. The hierarchical framework connects the population-level hyper-parameters to the observed data through the individual-galaxy-level parameters. According to the Bayes’ theorem, the posterior probability distribution of the hyper-parameters $u$ is given by

8 $$ p(u \mid\mathcal{D}) \propto p(\mathcal{D} \mid u) \ p(u), $$

where $\mathcal{D}$ is the dataset, $p(\mathcal{D} \mid u)$ is the likelihood, and $p(u)$ is the prior. For model parameters $w_{i}$ pertaining to individual galaxies, the above equation can be expressed as

9 $$ p(u \mid\mathcal{D}) \propto p(u) \prod_{i} \int\mathrm{d}w_{i} \ p( \mathcal{D}_{i} \mid w_{i}) \ p(w_{i} \mid u), $$

where $\mathcal{D}_{i}$ is the data from to the $i$-th individual lens galaxy. This approach can infer any property at the population level with the associated mean and scatter values. See, for example, Sonnenfeld and Cautun (2021) for a detailed presentation on the hierarchical framework with specific examples of hyper-parameters $u$.

### Incorporating Non-strong-Lensing Observables

Incorporating non-strong-lensing observables can be used to break the degeneracies in strong lensing analysis. For example, stellar kinematics data can be used to break the MSD (e.g., Romanowsky and Kochanek 1999; Treu and Koopmans 2002, 2004; Shajib et al. 2018; Birrer et al. 2020; Shajib et al. 2021; Tan et al. 2024), whereas spectroscopic stellar population analysis (e.g., Spiniello et al. 2011), weak lensing (e.g., Gavazzi et al. 2007; Sonnenfeld et al. 2018; Shajib et al. 2021), and microlensing (e.g., Schechter et al. 2014; Oguri et al. 2014) information can help mitigate the degeneracy between the stellar and dark matter distributions. Here, we briefly describe combining strong lensing with stellar kinematics (Sect. 3.5.1) and weak lensing (Sect. 3.5.2).

#### Combining Stellar Kinematics with Strong Lensing

Imaging observables probe the 2D mass distribution of the lens projected on the plane of the sky, whereas stellar kinematics probe its full 3D mass distribution. Thus, a combination of the two helps break the MSD to robustly constrain the mass distribution in galaxies. Although elliptical galaxies are triaxial, assuming spherical symmetry has been a standard practice for the case of a single aperture-integrated stellar velocity dispersion measurements (see Sonnenfeld et al. 2012, for a discussion on the impact of this assumption). Then, the stellar velocity dispersion is obtained by solving the spherical Jeans equation

10 $$ \frac{{\mathrm{d}} \left( l(r)\ \sigma_{\mathrm{r}}^{2} \right )}{{\mathrm{d}} r} + \frac{2 \beta_{\mathrm{ani}}(r)\ l(r) \ \sigma_{\mathrm {r}}^{2}}{r} = - l(r) \ \frac{{\mathrm{d}} \Phi}{{\mathrm{d}} r}. $$

Here, $l(r)$ is the 3D luminosity density of the stars, $\sigma_{\mathrm{r}}$ is the intrinsic radial velocity dispersion, and $\beta_{\mathrm{ani}}(r)$ is the anisotropy parameter relating $\sigma_{\mathrm{r}}$ with the tangential velocity dispersion $\sigma_{\mathrm{t}}$ given by

11 $$ \beta_{\mathrm{ani}}(r) \equiv1 - \frac{\sigma_{\mathrm{t}}^{2}}{\sigma_{\mathrm{r}}^{2}}. $$

By solving the Jeans equation, the line-of-sight velocity dispersion, which is the kinematic observable, is obtained as

12 $$ \sigma_{\mathrm{los}}^{2}(R) = \frac{2G}{I(R)} \int_{R}^{\infty} \mathcal{K}_{\beta} \left(\frac{r}{R} \right) \frac{l(r)\ M(r)}{r} \ {\mathrm{d}} r $$

(Mamon and Łokas 2005). Here, $M(r)$ is the 3D enclosed mass within radius $r$. The function $\mathcal{K}_{\beta}(u)$ depends on the parameterization of $\beta(r)$ (see Mamon and Łokas 2005 for specific forms of $\mathcal{K}_{\beta}(u)$ corresponding to different $\beta(r)$). Thus, the observed velocity dispersion in Eq. (12) can be written as a function of the lens model parameters as

13 $$ \sigma_{\mathrm{los}}^{2} = \frac{D_{\mathrm{s}}}{D_{\mathrm{ds}}}\ c^{2}\ J( \boldsymbol{\xi}_{\mathrm{mass}},\ \boldsymbol{\xi}_{\mathrm {light}},\ \beta_{ \mathrm{ani}},\ \lambda), $$

where $\boldsymbol{\xi}_{\mathrm{mass}}$ are the deflector’s mass model parameters, $\boldsymbol{\xi}_{\mathrm{light}}$ are the deflector’s light model parameters (Birrer et al. 2016). In this form, the function $J$ is independent of cosmology. It only depends on the lens model parameters $\{\boldsymbol{\xi}_{\mathrm{mass}},\ \boldsymbol{\xi}_{\mathrm {light}}\}$, the anisotropy profile $\beta_{\mathrm{ani}}(r)$, and the MST parameter $\lambda$. All the cosmological dependence of $\sigma_{\mathrm{los}}$ is contained in the distance ratio $D_{\mathrm{s}}/D_{\mathrm{ds}}$. To reproduce the observed velocity dispersion integrated within an aperture, the computed luminosity-weighted velocity dispersion needs to be blurred with the PSF $\mathcal{P}$ as

14 $$ \sigma^{2}_{\mathrm{ap}} = \frac{\int_{\mathrm{ap}} \left[ I(R)\ \sigma^{2}_{\mathrm {los}}(R) \right] * \mathcal{P} \ R \ \mathrm{d}R \mathrm{d}\theta }{ \int_{\mathrm{ap}} I(R) * \mathcal{P} \ R \ \mathrm{d}R \mathrm {d}\theta}, $$

where the ∗ symbol denotes the convolution operation. To obtain analytic solutions for specific choices of mass, light, and anisotropy profiles, see Koopmans (2006) for the case of power-law mass and light profiles with constant anisotropy, and Suyu et al. (2010) for the case with power-law mass profile, the Hernquist light profile (Hernquist 1990), and isotropic stellar orbits.

The constraints from the velocity dispersion measurement can be folded in the lens model posterior with a multiplicative likelihood term

15 $$ \mathcal{L}_{\mathrm{kin}} \propto\exp\left[ - \frac{(\sigma_{\mathrm{ap}}^{\mathrm{obs}} - \sigma_{\mathrm {ap}}^{\mathrm{model}})^{2}}{2 \sigma^{2}_{\sigma_{\mathrm {ap}}^{\mathrm{obs}}}} \right], $$

where $\sigma_{\sigma_{\mathrm{ap}}^{\mathrm{obs}}}$ is the uncertainty in the observed velocity dispersion $\sigma_{\mathrm{ap}}^{\mathrm{obs}}$. Whereas the imaging observables from strong lensing cannot constrain the MST parameter $\lambda$, the stellar velocity dispersion constrains $\lambda$ through the likelihood $\mathcal{L}_{\mathrm{kin}}$ (see Birrer et al. 2024, for a detailed discussion within a hierarchical Bayesian framework).

Although integrated velocity dispersion from long-slit spectra is the most commonly used kinematic observable in strong lensing studies, a few studies have also incorporated spatially resolved velocity dispersion, mainly from integral field unit (IFU) spectra (e.g., Barnabè and Koopmans 2007; Barnabè et al. 2011; Czoske et al. 2012; Spiniello et al. 2015).

#### Combining Weak Lensing with Strong Lensing

Weak lensing measures the excess shear quantity (${{\Delta}} \Sigma$) from tidal distortions of galaxies far away (≳10 arcsec) from the central lensing galaxy. Thus, weak lensing provides information on the mass distribution of the lensing galaxy’s outer region, that is, where the dark matter halo dominates. In contrast, strong-lensing observables provide information on the enclosed mass within the Einstein radius. Still, there remains a degeneracy between the luminous and dark matter fractions within the total enclosed mass. If a specific profile is assumed for the dark matter distribution, weak lensing data can break this degeneracy between luminous and dark component normalizations (Sonnenfeld 2018; Shajib et al. 2021).

The weak lensing information is often convenient or appropriate to be incorporated within the hierarchical framework, although there are examples of such combination without using a hierarchical framework (e.g., Gavazzi et al. 2007). Since the weak lensing signal from one single lensing galaxy does not usually have enough constraining power on the dark matter normalization, it is often required to stack weak lensing signals from a large sample of elliptical galaxies, which are not-necessarily lensing galaxies. If it is justified to assume that this large sample of elliptical galaxies and the lens galaxy sample under consideration are subsamples of the same parent population, then the weak lensing observables $\mathcal{D}_{\mathrm{weak}}$ and the strong-lensing observables $\mathcal{D}_{\mathrm{strong}}$ can be jointly considered in the posterior probability function of the population hyper-parameters $u$ as

16 $$ p(u \mid\mathcal{D}_{\mathrm{strong}}, \mathcal{D}_{\mathrm{weak}} ) \propto p( \mathcal{D}_{\mathrm{strong}} \mid u) \ p(\mathcal{D}_{\mathrm {weak}} \mid u) \ p(u). $$

Here, the strong lensing likelihood $p(\mathcal{D}_{\mathrm{strong}} \mid u)$ can then be expanded using the single system likelihoods similar to the product term in Eq. (9). Since from Bayes’ theorem, we have

17 $$ p(\mathcal{D}_{\mathrm{weak}} \mid u)\ p(u) \propto p(u \mid\mathcal{D}_{ \mathrm{weak}}), $$

it is also valid for numerical convenience to first obtain the posterior of $u$ from weak lensing observables only and then fold this posterior as the prior of $u$ in the hierarchical analysis with only strong-lensing data.

## Applications in Galaxy Properties and Evolution

This section describes what we can learn about galaxy structure and evolution using the lensing galaxy properties. Since strong-lensing galaxies are typically massive ellipticals, most of the strong-lensing studies in the field relate to this type of galaxy (Sect. 4.1–Sect. 4.3). However, we briefly discuss strong lensing by spiral galaxies at the end of this section in Sect. 4.4.

### Galaxy Mass Density Profile

All galaxies are believed to form and grow inside their dark matter halos. Thus, a massive galaxy’s total mass density profile comprises two components: the baryonic matter distribution, which includes stars and gas, and the dark matter distribution. As seen from the comparison of the observed galaxy luminosity function and the distribution of simulated dark matter halos (i.e., abundance matching, see Moster et al. 2010), the stellar-mass fraction decreases with mass, for the mass range of typical lensing galaxies. This is attributed to AGN feedback and is evident from simple comparisons of the stellar and total mass in lensing galaxies (Auger et al. 2009; Küng et al. 2018).

**Fig. 4:**
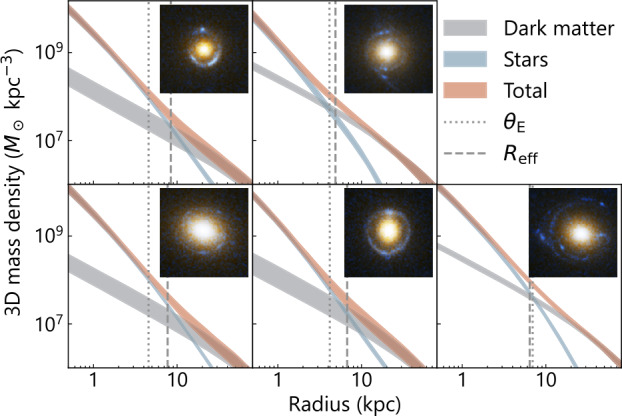
Examples of 3D mass distribution in dark matter and stars (i.e., baryons) in lensing elliptical galaxies (Shajib et al. 2021). The false-color image created from the *HST* imaging of each system is illustrated in the inset (*image credit: NASA/ESA, A. Bolton, and the SLACS team*). Shajib et al. (2021) combined this *HST* imaging data with the stellar kinematics and weak lensing information to decouple the stellar (teal) and dark matter (grey) components of the total mass density profile (red). Only five examples are illustrated here out of the 23 analyzed lenses from the SLACS sample. The vertical dotted and dashed lines mark the Einstein radius $\theta_{\mathrm{E}}$ and the half-light or effective radius $R_{\mathrm{eff}}$, respectively. In most cases, the total density profile is close to the power-law profile, with occasional deviations appearing near the center or far outside the Einstein radius

A power-law model $\rho(r) \propto r^{-\gamma _{\mathrm{pl}}}$ close to the isothermal case (i.e., $\gamma _{\mathrm{pl}}\sim2$) has been found to be sufficient to describe several lensing and non-lensing observables to the noise level; for example, from strong lensing only or in combination with stellar dynamics (Kochanek 1995; Treu and Koopmans 2004; Dye and Warren 2005; Koopmans et al. 2006, 2009; Barnabè et al. 2009; Auger et al. 2010b; Dutton and Treu 2014; Ritondale et al. 2019; Powell et al. 2022; Tan et al. 2024), from the combination of strong and weak lensing (Gavazzi et al. 2007), from stellar dynamics only (Bertin and Stiavelli 1993; Gerhard et al. 2001; Thomas et al. 2007; Tortora et al. 2014; Cappellari et al. 2015; Bellstedt et al. 2018; Derkenne et al. 2021), and from X-ray luminosity (Humphrey and Buote 2010). This phenomenon – that the total mass profile in ellipticals approximately follows the power law, whereas neither the baryonic nor the dark matter component individually follows the power law – is referred to as the ‘bulge–halo conspiracy’ (Treu and Koopmans 2004), similar to the ‘disk–halo conspiracy’ in spiral galaxies (van Albada and Sancisi 1986). Figure 4 illustrates the dark and luminous components constrained by combining kinematic and weak lensing information with strong lensing for five SLACS lens galaxies (Shajib et al. 2021). In most cases, the total density profile is very close to a power-law form, with deviations only being prominent far from the half-light radius. Galaxy formation simulations suggest that the close-to-isothermal nature of the total density profile originates from rearranging the mass distribution through collisionless accretion in gas-poor mergers (Johansson et al. 2009; Remus et al. 2013).

Analysis of the SLACS lenses finds the mean logarithmic slope $\langle\gamma_{\mathrm{pl}}\rangle= 2.08 \pm0.03$ with an intrinsic scatter of $0.16\pm0.02$ from a sample of 85 galaxy–galaxy lenses (Koopmans et al. 2009; Auger et al. 2010b). The median redshift of the SLACS lenses is $\langle z_{\mathrm{SLACS}} \rangle\simeq0.19$. The SIE lens model was adopted in this analysis to constrain $\theta_{\mathrm{E}}$ from the imaging data. Then, the power-law index $\gamma_{\mathrm{pl}}$ was obtained from the stellar velocity dispersion measured by the SDSS. Shajib et al. (2021) re-analyzed 23 systems from the SLACS sample with a power-law model instead of the SIE model to obtain the mean logarithmic slope $\langle\gamma_{\mathrm{pl}}\rangle= 2.08 \pm0.03$ with an intrinsic scatter of $0.13 \pm0.02$. Ritondale et al. (2019) analyzed 17 galaxy–galaxy lens systems from the BELLS GALLERY sample with a power-law mass model to find the average logarithmic slope $\langle\gamma_{\mathrm{pl}}\rangle= 2.00 \pm0.01$. The mean redshift of the BELLS GALLERY sample is approximately $\langle z_{\mathrm{BG}} \rangle\simeq0.5$.

**Fig. 5:**
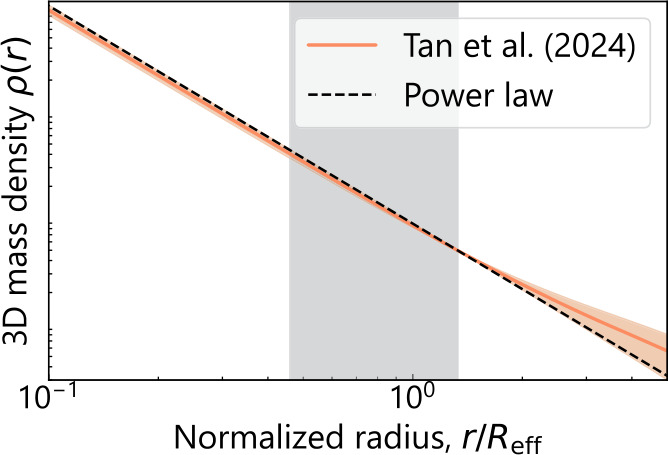
The shape of the mean 3D total mass profile for the lens galaxies from the SLACS and SL2S samples constrained by Tan et al. (2024, *cf.* Fig. 11 therein) from a joint lensing–dynamics analysis. The orange line shows the population mean with the shaded region representing the 68% (1$\sigma$) credible region. The dashed black line traces the fiducial power-law mass distribution. The vertical grey shaded region shows the 1$\sigma$ range of the Einstein radius distribution of the sample. For the dynamical modeling, these authors adopted a spatially constant stellar anisotropy profile, consistent with the spatially resolved velocity dispersion measurements of local elliptical galaxies (see references therein). The 3D mass density profile $\rho(r)$ along the vertical axis is normalized at a reference radius and thus has no units in this illustration. The population mean of the mass density profile is consistent with the power-law model within 1$\sigma$

Project Dinos (Tan et al. 2024) reanalyzed multi-band *HST* imaging for a sample of ∼50 lenses from the SLACS and SL2S samples and then combined the lensing constraints with the stellar kinematics to directly constrain any potential deviation from the power-law profile, where the deviation is parametrized with the internal MST parameter $\lambda_{\mathrm{int}}$ after correcting for the line-of-sight effects (i.e., the external convergence). The ‘internal’ MST is referred to as such to differentiate it from the effect of the external convergence that acts as an ‘external’ mass sheet. These authors find the power-law model is consistent with both the lensing and kinematic observables within $1\sigma$ for the baseline choice of spatially constant anisotropy profile that is informed by exquisite IFU kinematics of local elliptical galaxies (Fig. 5).

The surface mass in elliptical galaxies constrained from strong lensing can explain the origin of the so-called tilt of the fundamental plane, that is, the tight correlation between the effective radius, the effective surface brightness, and the central velocity dispersion (Ciotti et al. 1996). If the surface mass is used instead of the surface brightness, then the resulting mass plane (in place of the fundamental plane) is not tilted (Bolton et al. 2008a). This result implies that the tilt of the fundamental plane stems from the increase in the dark matter fraction with increasing velocity dispersion or dynamical mass (Auger et al. 2009).

In summary, the total mass profile in galaxies seems to be well described by a power law. However, there is also a recent indication of a potential departure from the power law suggested by the non-correlation between the logarithmic slopes from lensing-only and lensing–dynamics analyses (Etherington et al. 2023). The implications of this finding for the individual baryonic and dark matter components are discussed separately below.

#### Luminous (Baryonic) Mass Profile

In recent years, spatially resolved spectroscopic surveys of nearby ellipticals using IFUs and high-resolution *HST* imaging have considerably advanced our understanding of the structure and evolution of these galaxies (Cappellari 2016). Such detailed studies of intermediate redshift galaxies ($z\sim0.2-0.7$) using direct observations are impossible. However, the mass of galaxies acting as strong lenses at these redshifts can be mapped out in detail, thereby providing critical information about the progenitors of present-day galaxies and their evolution.

In the last decade, imaging and kinematic data have led to a revision of galaxy classification. The modern analyses classify ellipticals into fast and slow rotators, and those with and without central cores, resulting in four classes (Cappellari 2016; Krajnović et al. 2020). Massive ellipticals ($M\gtrsim10^{11}\mbox{M}_{\odot}$) in the local Universe tend to have small cores with size 0.02–0.50 kpc (Krajnović et al. 2020), that is, much smaller than the Einstein radius of typical lenses. In contrast, less massive ones appear to have cuspy central light profiles. Cores are believed to result from in-spiraling super-massive black hole (SMBH) binaries, which transfer their angular momentum outward to stars and leave a flatter density core at the center. The population of massive elliptical galaxies is also more likely to be composed of slow rotators, being also morphologically rounder than the lower mass fast rotator counter-part (e.g., Weijmans et al. 2014; van de Sande et al. 2017).

Although it is well known that the baryons and the dark matter do not follow the same radial profile, it is unknown *a priori* whether they have the same angular structure or not. Strong lensing allows us to compare the azimuthal distributions of the light and the total mass distributions, thus detecting any possible difference between the angular structures of the dark matter and the baryons. Several studies report a strong correlation in the ellipticity between the matter and light distributions (e.g., Koopmans 2006; Gavazzi et al. 2012; Sluse et al. 2012; Kostrzewa-Rutkowska et al. 2014). In contrast, some other studies only report weak or no correlation (e.g., Keeton et al. 1998; Ferreras et al. 2008; Rusu et al. 2016; Shajib et al. 2019, 2021). Some differences can be attributed to data quality, modeling procedure, or selection effects (Shajib et al. 2021). The major axes are usually well aligned (with position angle difference ≲10^∘^) between the mass and light distributions (e.g., Keeton et al. 1998; Kochanek 2022; Koopmans 2006; Treu et al. 2009; Sluse et al. 2012; Bruderer et al. 2016; Shajib et al. 2019, 2021). The cases where the major axes of the mass and light do not align within ∼10^∘^ also generally have large residual shear. These findings suggest that stellar orbits highly misaligned with the potential can only be sustained in non-isolated galaxies (indicated by the large residual shear if interpreted as originating from the presence of nearby galaxies), which is consistent with what is found in simulations (Heiligman and Schwarzschild 1979; Martinet and de Zeeuw 1988; Adams et al. 2007; Debattista et al. 2015). Although the stellar initial mass function (IMF) pertains to the luminous structure, we present the strong lensing results on the stellar IMF separately in Sect. 4.2.

#### Dark Matter Profile

Numerical simulations show that the dark matter halos and the baryonic mass within them initially follow the NFW profile before star formation begins. However, the baryonic gas has to cool down and fall inward for star formation to begin. The contraction in the baryonic matter deepens the gravitational potential. Thus, the dark matter distribution also contracts in response, for which the adiabatic contraction scenario can work reasonably well (Blumenthal et al. 1986; Cautun et al. 2020). In the process of adiabatic contraction of a spherical mass distribution, the initial radius $r_{\mathrm{i}}$ of a dark matter particle and its final radius $r_{\mathrm{f}}$ is related as

18 $$ r_{\mathrm{i}} \ M_{\mathrm{i}} (r_{\mathrm{i}}) = r_{\mathrm{f}} \ M_{\mathrm{f}} (r_{\mathrm{f}}), $$

where $M(r)$ is the enclosed 3D mass within radius $r$ (Blumenthal et al. 1986). However, numerical simulations find that dark matter does not fully respond to the baryonic infall according to the theoretical model of Blumenthal et al. (1986) (e.g., Gnedin et al. 2004; Abadi et al. 2010). Dutton et al. (2007) prescribe a formalism defining a halo response parameter $\nu$ to adjust the degree of contraction (i.e., the response to the baryonic infall) as

19 $$ r_{\mathrm{i}} \equiv\Gamma^{-\nu} (r_{\mathrm{f}})\ r_{\mathrm{f}}, $$

where $\Gamma(r_{\mathrm{f}}) \equiv r_{\mathrm{f}} / r_{\mathrm {i}}$ is the contraction factor. In this formalism, $\nu= 0$ corresponds to no contraction, and $\nu= 1$ corresponds to fully responsive contraction according to the model of Blumenthal et al. (1986). The simulations of Gnedin et al. (2004) point to $\nu\sim0.8$, and those of Abadi et al. (2010) to $\nu\sim0.4$ (Dutton and Treu 2014).

**Fig. 6:**
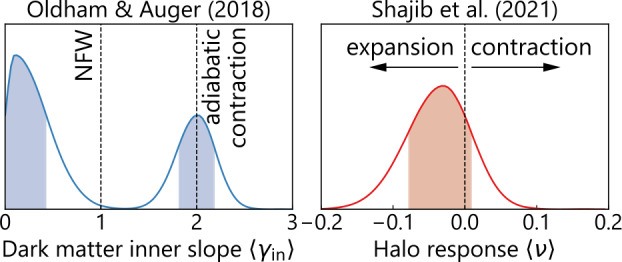
Results on the dark matter distribution from joint lensing–dynamics analyses. *Left-hand panel:* the probability density function for the sample mean of the dark matter inner logarithmic slope $\langle \gamma_{\mathrm{in}} \rangle$. This result was obtained from a hierarchical analysis performed on the lensing–dynamics data for 12 strong lenses. The probability distribution shows a bimodality, where one mode is consistent with the adiabatic contraction scenario (i.e., $\gamma_{\mathrm{in}} \approx2$) and the other mode is consistent with an expanded halo (i.e., $\gamma_{\mathrm{in}} \approx0$). *Right-hand panel:* the probability density function of the sample mean of the halo response parameter $\langle\nu\rangle$ obtained from a SLACS subsample of 23 lenses (Shajib et al. 2021). This result is consistent with no contraction or expansion from the regular NFW profile (i.e., $\nu=0$ marked by the vertical dashed line). Although these two results disagree, potential systematics stemming from different parametrizations of the adiabatic contraction or the stellar anisotropy profile are yet to be ruled out as the source of this discrepancy

Dye and Warren (2005) constrained the dark matter profile based on lensing analysis of one lens system and found the inner slope of the dark matter halo to be consistent with the NFW profile. Several studies have combined additional information, for example, stellar kinematics and weak lensing, with the strong-lensing data to decompose the dark matter and baryonic components from the total density profile constrained by strong lensing. Dutton and Treu (2014) adopted multiple dark matter contraction models with fixed $\nu$ to values between −0.5 and 1. These authors find that $\nu= 0$, that is, no contraction, best matches the data from the SLACS sample. Shajib et al. (2021) allowed for a fully variable $\nu$ parameter within −0.5 and 1 in their dark matter model. These authors find the average contraction in their sample to be $\langle\nu\rangle= -0.03^{+0.04}_{-0.05}$, which is consistent with no contraction and rules out the contraction results from simulations with high statistical significance (Fig. 6 right-hand panel). In contrast, Oldham and Auger (2018) find a cuspier inner logarithmic slope than the NFW profile for the majority of their sample of 12 lens systems, with a smaller subset having shallower ones, pointing to the impact of the environment in the evolution of these galaxies (Fig. 6 left-hand panel). However, the potential systematic dependence for all of the above results on modeling choices – for example, dark matter profile and anisotropy profile parameterizations – is yet to be investigated thoroughly.

### Stellar Initial Mass Function

When combined with ancillary data, strong lensing provides a method to infer the stellar mass-to-light ratio ($M_{\star }/L$; Treu et al. 2010), which can then be related to the low-mass end of the stellar IMF slope in the lensing galaxies (for a review, see Smith 2020). This is mainly because low-mass stars ($M_{\star}<0.5M_{\odot}$) contribute only by a few percent to the integrated light in the optical, but they give a much larger contribution to the mass (Conroy and van Dokkum 2012).

As described in Sect. 4.1, the total mass distribution in the lensing galaxy can be decomposed into the stellar and dark components by combining lensing and dynamical observables. Thus, the total stellar mass $M_{\star}^{\mathrm{LD}}$ can be obtained. An independent method to get the stellar mass $M_{\star}^{\mathrm{SPS}}$ of the galaxy is the stellar population synthesis (SPS) method applied on the lens galaxy’s photometric or spectroscopic data (Spiniello et al. 2011, 2012). The $M_{\star}^{\mathrm{SPS}}/L$ computed via SPS analysis depends on the choice of the IMF slope. In particular, a bottom-heavier IMF implies a larger $M_{\star}^{\mathrm{SPS}}/L$ because dwarf stars contribute more to the mass than light. Hence, the mismatch parameter, $\alpha_{\mathrm{IMF}} \equiv M_{\star}^{\mathrm{LD}} / M_{\star }^{\mathrm{SPS}}$, can be used to infer the lightness or heaviness of the IMF (Treu et al. 2010). For example, if a light IMF – such as the Chabrier IMF (Chabrier 2003) – is adopted in the SPS method, then $\alpha_{\mathrm{IMF}} \sim1$ would point to the Chabrier IMF being consistent with the lensing and dynamical observables. However, $\alpha_{\mathrm{IMF}} \sim2$ would indicate that the IMF in the lens galaxies is bottom-heavier (i.e., characterized by a larger number of dwarf stars), with a slope more similar to that of a Salpeter IMF (Salpeter 1955), or even steeper. Note that $M_{\star}^{\mathrm{LD}}$ may depend on lens modeling assumptions, such as the choice of dark matter density profile, the stellar mass-to-light ratio being spatially constant or varying, and the assumed anisotropy profile of the stellar orbits in the dynamical modeling (e.g., see the discussions in Sonnenfeld et al. 2018, 2019).

Although the IMF slope and the low-mass cutoff are degenerate with respect to the lensing data, both can be constrained when combined with dynamics and stellar population analysis. Barnabè et al. (2013) show this by studying two strong lenses from the X-Shooter Lens Survey (Spiniello et al. 2011) for which both *HST* imaging (for precise lens modeling) and X-Shooter spectra (for stellar population analysis) are available. Chromatic, microlensing-induced flux anomalies in a galaxy–quasar strong lens can also be used to constrain the stellar IMF (Schechter et al. 2014). This technique is described in Vernardos et al. (2024), which discusses the theory and applications of microlensing.

**Fig. 7:**
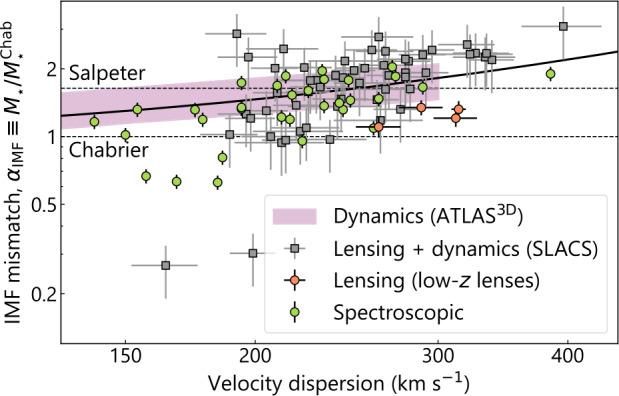
Measurements of the stellar IMF from various probes. The pink shaded region traces the 1$\sigma$ intrinsic scatter of the fitted relation from dynamical constraints of the ATLAS^3D^ sample (Cappellari et al. 2013; McDermid et al. 2014). The grey points are based on joint lensing–dynamics analysis for a subsample of the SLACS lenses (Posacki et al. 2015). The orange points are lensing-only measurements from a sample of low-redshift ($z=0.031$–0.066) lenses; 3 from the SINFONI Nearby Elliptical Lens Locator Survey (SNELLS) and one discovered from a search on the publicly available MUSE data (Smith et al. 2015; Collier et al. 2018). The green points show the fully spectroscopy-based measurements for a sample of 34 elliptical galaxies from Conroy and van Dokkum (2012). The two horizontal dashed lines mark the values expected for Salpeter and Chabrier IMFs. The solid black line illustrates the dependency of the IMF on the velocity dispersion, fitted by Posacki et al. (2015) using the ATLAS^3D^ and the SLACS samples

Whereas the IMF within the Milky Way is light – that is, consistent with the Chabrier IMF regardless of the stellar population age and environment (Chabrier 2003; Bastian et al. 2010) – the majority of strong lensing studies on elliptical galaxies report consistency with a heavier IMF (e.g., Spiniello et al. 2011; Sonnenfeld et al. 2012; Oldham and Auger 2018). We note, however, that the stellar IMF is degenerate with the choice of the dark matter density profile in most of these studies (Auger et al. 2010a). For instance, the SLACS analysis – by combining lensing and dynamics – finds the IMF in elliptical galaxies at mean redshift $\langle z \rangle\sim0.2$ to be consistent with the Salpeter IMF, that is, $\alpha_{\mathrm{IMF}} \sim2$ (see Fig. 7; Treu et al. 2010, also, Grillo et al. 2009). This result is reproduced with more flexible models for the same SLACS systems or a subset of them (Auger et al. 2010b; Shajib et al. 2021). This is also in agreement with the more general findings, based on dynamics or SPS analysis only, that the IMF is bottom-heavier for more massive galaxies in general (Fig. 7; Cappellari et al. 2012; La Barbera et al. 2013; Spiniello et al. 2014).The general picture is that the low-mass end of the IMF might not be universal across all galaxies, as generally assumed in the last thirty years. However, a consensus on the physical mechanisms responsible for its variation has not yet been reached. According to theoretical work (e.g., Hopkins 2013; Chabrier et al. 2014), high density, temperature, and turbulence of the gas are key parameters that drive the fragmentation of molecular clouds. Higher density and temperature make the fragmentation easier, forming more dwarf stars, that is, a bottom-heavier IMF.

In contrast with the studies mentioned above on massive, local elliptical galaxies, Sonnenfeld et al. (2019) find $\log_{10} \alpha_{\mathrm{IMF}} = -0.04 \pm0.11$ with respect to the Chabrier IMF in the SPS method for a sample of strong lensing galaxies at $z \sim0.6$, which is in tension with Shetty and Cappellari (2014) that reports consistency with a Salpeter IMF for a galaxy at $z\sim1$ from a dynamical analysis. However, allowing spatial gradients in the $M_{\star}/L$ can alleviate this tension (Sonnenfeld et al. 2018, 2019). Indeed, a radial gradient in the $M_{\star}/L$, or equivalently a radially varying IMF, has been reported by studies based on the SPS method applied on local massive ellipticals (Martín-Navarro et al. 2015b; van Dokkum et al. 2017; Sarzi et al. 2018; Barbosa et al. 2021a,b). These authors find that the central region within ∼2 kpc has a heavy IMF (even super-Salpeter), and the IMF in the outer regions gradually becomes light (i.e., Milky-Way-like). The current belief is that the IMF is bottom-heavy for stars formed very early in cosmic time via a quick and violent SF burst. These stars usually form a ‘red nugget’ ($z\sim 2$, Damjanov et al. 2011; Oldham et al. 2017): an ultra-compact red-and-dead massive core. Then, with a second and more time-extended phase, red nuggets merge, interact with other structures in the Universe, and accrete gas. This process causes a growth in size up to a factor of ∼5, and only slightly in mass, transforming them into giant local, massive ellipticals. Depending on the merger history of each single galaxy, the red nugget can remain almost untouched in the innermost region, dominating the light there. In this case, this region would have a bottom-heavy IMF. This seems to be the case for NGC 3311, the central galaxy in the Hydra cluster (Barbosa et al. 2021a), M87 (Sarzi et al. 2018), and many other very massive low-$z$ elliptical galaxies. However, the red nugget can also be destroyed or contaminated by accreted or lately formed stars. In that case, one would measure a Milky-Way-like IMF, which is the characteristic for stars formed later on and through more time-extended star formation channels. This ‘two-phase formation scenario’ (Naab et al. 2009) is also supported by the discovery of ‘relic galaxies’ (Trujillo et al. 2014; Spiniello et al. 2021), the local counterparts of red nuggets that somehow wholly missed the size-growth and evolved passively and undisturbed across cosmic time. The IMF for these peculiar and rare objects has been measured to be steep everywhere up to at least one effective radius (Ferré-Mateu et al. 2017). Finally, simulations have lately shown that not all elliptical galaxies formed via this two-phase formation scenario (e.g., Pulsoni et al. 2021), although this becomes more and more common with increasing stellar mass.

The finding of the Salpeter IMF from a combination of lensing and dynamics in the near-by Universe is consistent with this scenario, as strong-lensing information is sensitive to the galaxy’s inner region (typically ≲6 kpc). However, at higher redshift, lensing probes larger and larger regions, which explains the results presented in Sonnenfeld et al. (2019). A radially decreasing $M_{\star}/L$ also explains or alleviates the reported tension between lensing-based studies themselves (Sonnenfeld et al. 2018, 2019; Shajib et al. 2021).

Furthermore, studies of few local massive lenses for which the Einstein radius is much smaller than the effective radius, and hence where the stellar mass dominates the lensing inference, indicate that bottom-heavy IMFs are excluded by lensing (Ferreras et al. 2010; Smith et al. 2015; Leier et al. 2016). Allowing for a variable cut-off on the low-mass end of the IMF can reconcile the $M_{\star}/L$ measurement from strong lensing of Smith et al. (2015) with the IMF-sensitive absorption line measurements of Conroy and van Dokkum (2012). Still, the discrepancy with other lensing–dynamics measurements remains.

In conclusion, the currently preferred scenario sees the majority of massive galaxies having a bottom-heavy IMF in their innermost region, where the pristine stellar population dominates that formed at $z>2$ through a star formation burst, while stars in outskirt are distributed by a Milky-Way-like IMF. However, depending on the single galaxy’s detailed merger tree and cosmic evolution, the IMF can differ from system to system.

In the future, IFU-based stellar kinematics and population analysis of strong lensing galaxies in combination with lensing constraints can shed light on the presence or absence of IMF variations and spatial gradients in ellipticals at intermediate redshifts. However, to properly track any evolution in the IMF properties of elliptical galaxies across redshift, it would be essential to mitigate systematic impacts through a uniform choice of models and to account for selection differences between samples.

### Constraints on the Very Central Densities and SMBH Mass from Central Images

Gravitational lensing theory predicts that the number of multiple images must always be odd. In systems with three (or five) images, two (or four) are formed roughly at the Einstein radius from the lens center, which is  for galaxies. The odd 3rd (or 5th) image is formed very near the center of the lens. It is always demagnified, usually significantly, and superimposed on the light of the lensing galaxy, making it hard to detect. Since the demagnification of the central image depends on the central density profile, the detections (or the lack thereof) of the central image can constrain or put an upper limit (or lower limit) on the steepness of the inner density profile or the SMBH mass (e.g., Winn et al. 2004), with the degeneracy between the two broken with stellar light distribution informing the stellar mass profile (e.g., Wong et al. 2015; Tamura et al. 2015).

Most existing searches for central images of strongly lensed quasars (given that they are much brighter sources) rely on optical, or radio wavelengths. The radio wavelengths are most favorable as the lensing galaxy is generally transparent in that range, but these investigations are limited by the fact that quasars are usually radio-quiet. Out of ∼200 doubles discovered to date, only two have observed central images where the lens is a single galaxy:^5^ PMN J1632−0033, with the central image demagnified by a factor of 0.004 or 6 magnitudes compared to the brightest image (Winn et al. 2004), and PKS 1830−211, with the central image demagnified by a factor of 0.007 or 5.4 magnitudes (Muller et al. 2020). No reliable detection exists of the ∼50 known quads with a single lensing galaxy. Upper limits have also been placed. For example, Quinn et al. (2016) place an upper limit of $\sim10^{-4}$ on the magnification of the central image with respect to the brightest visible image in the double B1030+074.

Upper limits on the flux of the central image have also been placed at X-ray wavelengths for several quads and doubles. The stacking of X-ray monitoring data allows effective exposures of several hundreds of kilo-seconds without any contamination from the lens. However, the data are still too shallow to strongly constrain the lens galaxy’s inner density profile. One of the deepest upper limits has been achieved for HE 0435−1123 (e.g., Chen et al. 2012; Guerras et al. 2017).

Detection of central images, or the lack thereof, has been used to place constraints on the central mass density of the lensing galaxies and the mass of the central SMBH (Mao et al. 2001; Rusin and Ma 2001; Wong et al. 2015; Tamura et al. 2015; Quinn et al. 2016; Perera et al. 2023). Even without a central image detected, a lensed image that is sufficiently close to the center can be used to measure the SMBH mass. For example, such an SMBH mass ($M_{\mathrm{SMBH}} = 3.27\pm2.12 \times10^{10}$ M_⊙_; 3$\sigma$ confidence limit) was measured using the image at ∼1 kpc distance from the center in Abel 1201 ($z_{\mathrm{lens}} = 0.169$), providing the first lensing-based measurement of an SMBH mass with limits placed on both sides (Nightingale et al. 2023). Additionally, Millon et al. (2023) demonstrated the usefulness of strong lensing *by* a quasar (SDSS J0919+2720, shown in Fig. 1) to measure its host galaxy mass to robustly probe the SMBH–host mass relation, where the SMBH mass was measured through conventional methods based on spectroscopic data.

For other kinds of SMBHs, prospects for detecting binary SMBHs due to their lensing effects are discussed in Li et al. (2012), Hezaveh et al. (2015). Free-floating SMBHs, which could be of primordial origin or formed through co-evolution with their previous host galaxies, can be detected through the kinks they produce on razor-thin arcs with sub-mas width and resolved in radio observations (Banik et al. 2019). The detection (or the lack thereof) of such free-floating SMBHs can place constraints (or upper limits) on their mass density and fractional contribution to the dark matter.

### Spiral Galaxies

Due to their lower mass and presence of a disk, spiral galaxies have a substantially lower lensing cross-section than elliptical ones (Keeton and Kochanek 1998). Only a handful of spiral galaxies lensing quasars have been discovered to date. The most well-known and the first example of such a system is the Einstein Cross (2237+0305), which is lensed by the bulge of a nearby spiral galaxy (Huchra et al. 1985). Targeted searches for galaxies lensed by a spiral have been carried out, allowing the discovery of several dozens of systems (Treu et al. 2011, and reference therein). Complications in studying those systems arise from the dust and the disk mass component. Correcting for the reddening by dust is needed to model extended lensed images, which rely on conserving the surface brightness between the lens and the source plane. On the other hand, the disk component yields strong discontinuities in the gravitational potential and needs to be explicitly modeled using, for example, an exponential mass density profile. Whereas disk-like features can yield flux anomalies in lensed quasars (Hsueh et al. 2016, 2017; Gilman et al. 2017), they can be disentangled from the dust using multi-band data (Möller et al. 2003). Once these ingredients are accounted for, the combination of kinematics and lensing information can be used to break the degeneracy – that exists in dynamical studies alone – between the disk and halo components (Maller et al. 2000; Dutton et al. 2011; Suyu et al. 2012).

## Applications in Cosmology

In this section, we present applications of galaxy-scale lens systems to measure cosmological parameters without using time-delay information. Measurement of the Hubble constant ($H_{\mathrm{0}}$) and other cosmological parameters based on the time delays are discussed in detail in Birrer et al. (2024).

Here, we briefly establish some necessary definitions for use in this section. More detailed explanations of the cosmological connection with strong lensing formalism are given in Saha et al. (2024). The angular diameter distance between two objects at redshifts $z_{1}$ and $z_{2}$ (with $z_{1}< z_{2}$) for a flat universe is given by

20 $$ D_{\mathrm{ang}}(z_{1}, z_{2})=\frac{c}{H_{0} (1+z_{2})} \int_{z_{1}}^{z_{2}} \frac{\mathrm{d}z'}{E(z')}, $$

where $E(z) \equiv H(z)/H_{0}$ is the dimensionless Friedman equation given by

21 $$ E(z) = \sqrt{{\Omega_{\mathrm{m}}} (1+z)^{3} + {\Omega_{\mathrm{r}}} (1+z)^{4} + {\Omega}_{\mathrm{de}} (1+z)^{3(1+w_{\mathrm{de}})}}. $$

Here, ${\Omega_{\mathrm{m}}}$, ${\Omega_{\mathrm{r}}}$, and ${\Omega}_{\mathrm{de}}$ represent the matter, radiation, and dark energy density parameters, respectively, at $z=0$. The parameter $w_{\mathrm{de}}$ is the equation-of-state parameter of the dark energy given by $w_{\mathrm{de}} \equiv p_{\mathrm{de}}/\rho_{\mathrm{de}} c^{2}$, where $p_{\mathrm{de}}$ and $\rho_{\mathrm{de}}$ denote the pressure and density of the dark energy, respectively. In the $\Lambda$CDM model, $w_{\mathrm{de}} = -1$ is assumed, that is, the dark energy density stays constant through cosmic time. The $w$CDM model is one natural extension of the $\Lambda$CDM model, where $w_{\mathrm{de}} \neq-1$ is allowed; however, $w_{\mathrm{de}} < -1/3$ should still be satisfied to reproduce an accelerated Universe.

In the following subsections, we discuss methods to estimate cosmological parameters (primarily, $\Omega_{\mathrm{m}}$, $\Omega_{\mathrm {de}}$, and $w_{\mathrm{de}}$) using multiple-source-plane lenses (Sect. 5.1), using the stellar kinematics of strong lenses (Sect. 5.2), and using galaxy–galaxy lensing statistics (Sect. 5.3).

### Utilizing Multiple Sources at Different Redshifts

Strong lens systems with multiple sources (i.e., compound lenses) at different redshifts can be used as cosmographic probes. Currently, only a tiny sample of galaxy-scale compound lenses are known (e.g., Lewis et al. 2002; Gavazzi et al. 2008; Collett and Smith 2020), but at the *HST* snapshot depth they are expected to occur in about 1% of galaxy-scale strong lenses (Gavazzi et al. 2008). Indeed, if one were to stare deeply at any single plane lens, other sources would almost inevitably be discovered, as is spectacularly demonstrated by the discovery of a third source (a $z \approx6$ Lyman-$\alpha$ emitter) behind the Jackpot lens in deep Multi Unit Spectroscopic Explorer (MUSE) data from the Very Large Telescope (Collett and Smith 2020). Forthcoming surveys are expected to discover $\mathcal{O}(1000)$ compound lenses.

Since the Einstein radius is a function of the lens mass and the cosmological distances, the ratio of Einstein radii in a compound lens with sources at two or more redshifts is independent of the mass (Gavazzi et al. 2008; Collett et al. 2012). In practice, the method also requires a complete understanding of the lens density profile and additional lensing by the source galaxies and other perturbing masses along the line of sight.

For a two-source-plane system with one primary lens, the lens equation can be written as

22 $$\begin{array}{rl} & y=x-\beta_{12}\alpha_{d}(x),\\ & z=x-\alpha_{d}(x)-\alpha_{s1}\left(x-\beta_{12}\alpha_{d}(x)\right), \end{array}$$

where $\mathbf{x}$ are positions on the image plane, $\mathbf{y}$ and $\mathbf{z}$ are the unlensed positions of the first and second source, respectively, $\alpha_{d}$ is the deflection caused by the primary lens, $\alpha_{s1}$ is the deflection caused by the closer of the two sources, and $\beta_{ij}$ is the cosmological scaling factor

23 $$ \beta_{ij} = \frac{D_{ij} D_{\mathrm{s}}}{D_{j} D_{i \mathrm{s}}}. $$

For realistic redshifts and cosmologies, $\beta_{ij}$ is sensitive to the matter density parameter $\Omega_{\mathrm{m}}$ and the equation-of-state parameter $w_{\mathrm{de}}$ but has no dependence on the Hubble constant $H_{\mathrm{0}}$.

**Fig. 8:**
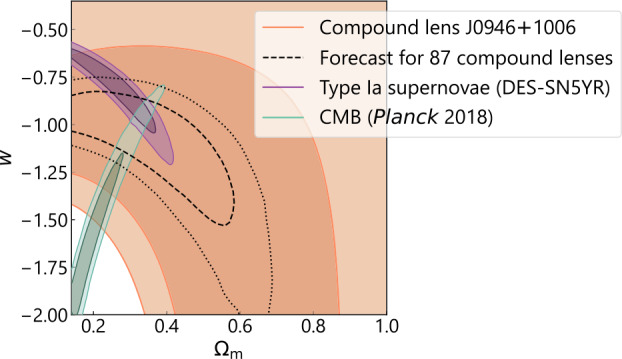
Cosmological parameters constrained from the compound lens system SDSS J0946+1006 (shown in Fig. 2). The 68% and 95% credible regions on the $\Omega _{\mathrm{m}}$–$w_{\mathrm {de}}$ plane from this compound plane system are shown in orange (Collett and Auger 2014). The black dashed and dotted contours illustrate the forecasted 68% and 95% credible regions, respectively, for a sample of 87 compound lenses to be discovered by the Rubin Observatory LSST (Sharma et al. 2023). The purple and emerald contours are the constraints from the DES Type Ia supernovae sample (DES Collaboration 2024) and the CMB (Planck Collaboration 2020), respectively, illustrating the complementarity of compound lenses to these probes

Amongst galaxy-scale compound lenses, only the Jackpot lens (Gavazzi et al. 2008), shown in Fig. 2, has been used to precisely constrain cosmology since this system has a favorable redshift configuration – other compound lenses have multiple sources at similar redshifts, thus having $\beta\approx1$ regardless of the cosmology. Collett and Auger (2014) modeled the *HST* imaging performing a pixellated reconstruction of both sources to make a 1.1% measurement on $\beta^{-1}$. Converting this into constraints on the dark energy, this single compound lens with a cosmic microwave background (CMB) prior from Planck Collaboration (2014) constrains $w_{\mathrm{de}}$ to 0.2 precision. With hundreds of compound lenses expected in the Rubin Observatory Legacy Survey of Space and Time (LSST) and the *Euclid* surveys, constraints on both $w_{\mathrm{de}}$ and its redshift derivative are expected to be comparable with established cosmological probes (Fig. 8; Sharma et al. 2023).

### Utilizing Stellar Kinematics of Single Source Lenses

The enclosed projected mass inside the Einstein radius is independent of the mass profile (Schneider et al. 1992). Similarly, the speeds of the stars within a galaxy are sensitive to the total mass enclosed within their orbits. Whilst we cannot measure the speeds of individual stars in a lens, we can measure the velocity dispersion of the ensemble. For an SIS lens with stars on isotropic orbits, these two quantities are related by

24 $$ \theta_{\mathrm{E}}= 4 \pi\left( \frac{\sigma_{\mathrm{los}}}{c} \right)^{2} \frac{D_{\mathrm{ds}}}{D_{\mathrm{s}}}, $$

where ${\sigma_{\mathrm{los}}}$ is the velocity dispersion of the deflector. Ofek et al. (2003) estimated that deviations from isothermality and orbital isotropy can cause the observed velocity dispersion to differ from Eq. (24) by up to 20%.

Equation (24) becomes far more complicated if the density profile is not isothermal or the orbits of the stars are not isotropic, but the fundamental relationship remains that one can constrain the distance ratio $d^{\mathrm{obs}} \equiv D_{\mathrm{ds}}/D_{\mathrm{s}}$ using the observed Einstein radius $\theta_{\mathrm{E}}$ and observed velocity dispersion. Thus, a sample of single-source lenses with measured $\sigma_{\mathrm{los}}$ can be used to estimate cosmological parameters (Grillo et al. 2008) by maximizing the likelihood function

25 $$ \mathcal{L}\left(\boldsymbol{\Theta} \mid\mathcal{D} \right) \propto \exp\left[ -\frac{1}{2} \sum_{i=1}^{N_{\mathrm{SL}}} \frac{ \left[ d^{\mathrm{th}}_{i}\left(z_{\mathrm{d}}, z_{\mathrm{s}}; \boldsymbol{{\Theta} }\right) - d^{\mathrm{obs}}_{i}({\theta _{\mathrm{E}}}_{,i}, \sigma_{{\mathrm{los}}, i})\right]^{2} }{ (\delta d^{\mathrm{{obs}}}_{i})^{2}} \right], $$

where $\boldsymbol{\Theta}$ is a set consisting of the free parameters in the assumed cosmological model and the free parameters that describe the density profile and anisotropy profile of the lenses, $\mathcal{D}$ is the data, $N_{\mathrm{SL}}$ is the number of lens systems, and $\delta d_{i}^{\mathrm{{obs}}}$ is the uncertainty of each $d_{i}^{\mathrm{obs}}$, which depends on the $\sigma_{{\mathrm {los}}, i}$ and ${\theta_{\mathrm{E}}}_{,i}$ uncertainties.

Similar to the multiple-source lens systems described in Sect. 5.1, this method is also independent of $H_{\mathrm{0}}$. If the cosmological parameters and the lens population parameters are inferred simultaneously for a large sample of lenses (e.g., $N_{\mathrm {lens}}\sim$10 000) discovered by the Rubin Observatory LSST and *Euclid*, it will be possible to achieve very competitive precision with other probes such as Type Ia supernovae and the CMB (Li et al. 2024).

The primary systematics in this method can potentially arise from these assumptions: (i) the measured $\theta_{\mathrm{E}}$ is independent of the choice of the lens mass profile (e.g., Cao et al. 2015), and (ii) the measured line-of-sight velocity dispersion is equal to that for an SIS profile. Recent modeling methods provide robust $\theta_{\mathrm{E}}$ measurement within a few percent regardless of the mass profile choice (Birrer 2021). Treu et al. (2006) argue that $\sigma_{\mathrm{los}}\simeq\sigma_{\mathrm{SIS}}$ for the lens elliptical galaxies with velocity dispersion in the range 200–300 km s^−1^. These results are further confirmed by analyzing other samples (Bolton et al. 2006; Auger et al. 2010b).

Ultimately, the only way to move forward with lensing and dynamics as a precision cosmological probe is to simultaneously infer the astrophysical parameters of the lens population and the cosmological parameters. By building up a sample of many lenses it should be possible to investigate how the Einstein radius grows with source redshift regardless of the underlying density profile of strong lenses. Exploiting the fact that lenses at the same redshift can be expected to be somewhat self-similar, Li et al. (2024) showed that with 10 000 lenses, it is possible to disentangle lens population properties and cosmological parameters. These authors assumed that lenses have the same intrinsic scatter as Auger et al. (2010b) found for the SLACS lenses. The method is fundamentally limited by how self-similar lenses are, and if their properties evolve with redshift.

### Utilizing Galaxy–Galaxy Lensing Statistics

The statistics of strong lensing were initially expected to be powerful for probing the cosmological parameters (Fukugita et al. 1990). At the most basic level, matter clusters can collapse to the densities required to form strong lenses, whereas the cosmological constant does not cluster and cannot form lenses. Therefore, it was expected that lensing rates should be suppressed for larger values of $\Omega_{\Lambda}$. Further information is contained in the Einstein radius distribution and the lens and source redshift distributions. In practice, this topic has fallen out of fashion due to the cosmological sensitivity being overwhelmed by astrophysical uncertainties of the unlensed source population, the lens discovery selection function, and the lensing properties of typical galaxies (e.g., Mitchell et al. 2005; Chae 2010).

## Open Problems and Future Outlook

In this section, we discuss the current open problems that are expected to be tackled in this decade: the selection function (Sect. 6.1), the self-similarity assumption (Sect. 6.2), degeneracies in strong-lensing (Sect. 6.3) and non-strong-lensing observables (Sect. 6.4), and comparison with galaxy simulations (Sect. 6.5). We also provide future outlooks on these issues whenever appropriate.

### Selection Function

**Fig. 9:**
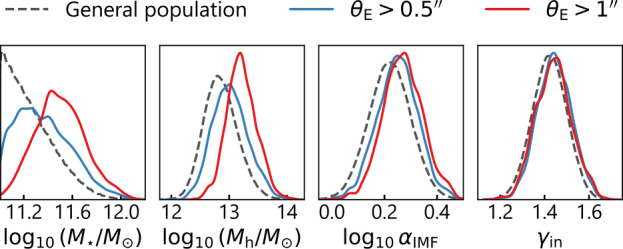
Impact of strong lensing selection function, estimated by Sonnenfeld et al. (2023), on various galaxy properties: from left to right, stellar mass $M_{\star}$, halo mass $M_{\mathrm{h}}$, IMF mismatch parameter $\alpha_{\mathrm{IMF}}$ with $\alpha_{\mathrm{IMF}} = 1$ corresponding to the Chabrier IMF, and the dark matter’s inner logarithmic slope $\gamma_{\mathrm{in}}$. The grey dashed line shows the distribution of the general population of galaxies. The blue and red lines show the distributions of galaxy–galaxy strong lenses with  and , respectively. Strong lenses are expectedly biased toward more massive galaxies. The mean IMF mismatch parameter in strong lensing galaxies is biased by 10% from the general population, and the mean inner logarithmic slope is biased by 5%

Although strong lensing is a powerful probe to study galaxy properties, the lensing phenomenon is a rare occurrence requiring a serendipitous alignment of two line-of-sight objects separated by a large cosmological distance. Thus, samples of strong lensing galaxies inherently occupy a tiny fraction of the population of all galaxies. When strong lensing studies aim to infer properties of the general population of galaxies based on such a small fraction of galaxies, the lens sample’s selection function must be considered. The strong lensing samples are inherently biased towards lensing galaxies that are more massive and concentrated (Mandelbaum et al. 2009; Sonnenfeld et al. 2023). Although triaxial galaxies with the major axis more aligned along the line of sight also have larger lensing cross-sections, interestingly for a given mass and shape, the effect of viewing angle does not affect the selection function when averaged over (Mandelbaum et al. 2009, we note that this study only considered point sources). Sonnenfeld et al. (2023) estimate that the mean of the IMF mismatch parameter $\alpha_{\mathrm{IMF}}$ measured from a sample of lens galaxies is only biased by 10% and the mean of the inner slope of the dark matter by 5% (Fig. 9). These bias levels are dependent on the completeness in the Einstein radius distribution of the lens sample but independent of the source properties, with the galaxy–galaxy lenses and galaxy–quasar lenses having the same levels of bias.

The lens samples to date often had highly complex selection functions, largely due to the selection or discovery procedure being highly tuned to maximize the number of discovered systems. As a result, treatment of selection function on actual lens samples has been rare except for a handful of studies (Arneson et al. 2012; Sonnenfeld et al. 2015, 2019). For the ideal case of a known selection function, Sonnenfeld (2022) provides the formally correct solution to account for selection effects in the lens samples. However, several technical challenges still remain to implement this formal solution on real samples, for example, the requirement to well characterize the lens-detection efficiency of the survey and also the efficiency in obtaining follow-up spectroscopy if that was taken into account for sample selection. The treatment of the selection effect with parametric functions by Sonnenfeld et al. (2019) can be considered as an approximation to this formal solution. To keep the selection function easily treatable, it can be advisable to pre-emptively mitigate the complexity of the selection procedure. Alternatively, it can be possible to form a subsample of lens systems that has a well-characterized selection function from a much larger sample of discovered systems, for example, those discovered by current and future surveys such as the *Euclid*, the Rubin Observatory LSST, and the *Roman Space Telescope* (Collett 2015; Sonnenfeld 2022).

### Assumption of Self-Similarity

When hierarchically inferring population properties of lenses by combining multiple strong lensing systems (Sect. 3.4), it is crucial to describe and quantify potential differences between subsets of the considered sample for accurate population-level inference. Differences in the population may arise from the sample selection due to search criteria and techniques (Sect. 6.1) or intrinsic differences in the sources. Such differences in secondary selection might impact and bias population-level constraints when assuming that two different samples can be described with an identical underlying population. For example, Birrer et al. (2020) present a hierarchical analysis under the assumptions that the same population level parameters describe the SLACS and the Time-Delay COSMOgraphy (TDCOSMO) lenses to constrain the mass density profiles of the time-delay lenses better. There are different ways to mitigate such assumptions. The first is to select a purified sample of lenses as self-similar as possible when performing hierarchical analyses, also suggested in Sect. 6.1. However, although the currently small number of known lenses will increase through future surveys and enable this approach, it is still unclear in which parameter space these lenses can be considered self-similar and whether there are important latent variables to consider. The second way is to model and describe differences between populations on a first-principle level, folding in differential selection effects and other aspects into the analysis (as stated in Sect. 6.1).

### Degeneracies in Strong-Lensing Observables

The lensing data’s ability to constrain the lens’s radial structure, particularly in disentangling dark and luminous mass components, strongly depends on the data quality. As discussed in Saha et al. (2024), the modeling of point-source astrometry primarily encodes information on the lens’s quadrupole moment. Still, it provides limited constraints on the monopole (the total mass). This, however, does not mean that any monopole model combined with a quadrupole component will accurately reproduce a set of lensed image positions. For some choice of the monopole, extreme or unphysical values of the quadrupole may be required, naturally excluding some mathematically correct solutions. This explains why, for example, a single-component, constant mass-to-light ratio model generally yields large shear amplitudes to reproduce the observed astrometry of lensed systems to high accuracy (e.g., Sluse et al. 2012).

While extended lensed images may provide detailed azimuthal information and allow one to constrain the ratio of radial magnifications at different galactocentric distances, their effective constraint on the density profile remains sensitive to the MSD (e.g., Sonnenfeld 2018). While the MSD provides mathematically large leverage to modify the results, its impact may remain in practice generally small, with a typical change on the total density profile that may not exceed a few tens percent. The prior on the mass profile (i.e., choice of mass distribution families or a free-form model with some regularization) may further limit the impact of degeneracies. One may, however, need to be careful when choosing a mass distribution, as a model that is too rigid compared to the true mass density may yield biased posteriors or underestimated parameter uncertainties (e.g., Sonnenfeld 2018; Kochanek 2021). Finally, it is important to note that point images with measured time delays limit the impact of degeneracies in lensing-only observables for an assumed cosmology (Saha and Williams 2001; Kochanek 2002).

### Degeneracies in Non-strong-Lensing Observables

The modeling of stellar kinematics data requires an assumption on the anisotropy profile of stellar orbits. Integrated velocity dispersions obtained from single-slit spectra cannot constrain the anisotropy profile, which leads to the so-called mass–anisotropy degeneracy (Treu and Koopmans 2002) — typically adopted anisotropy profiles are either isotropic or the Osipkov–Merritt profile (Osipkov 1979; Merritt 1985a,b). Whereas the isotropy assumption does not entail any free parameter, the Osipkov–Merritt profile depends on a scale radius. Due to the mass–anisotropy degeneracy, the posterior of the anisotropy scale radius is dominated by the adopted prior (e.g., Shajib et al. 2018). Particular choices of the anisotropy profile and the associated prior may lead to systematic differences between studies involving strong lensing and kinematics data. For example, Sonnenfeld et al. (2018) find the existence of a mass-to-light ratio gradient in the SLACS lenses assuming isotropic orbits, whereas Shajib et al. (2021) find consistency with a constant mass-to-light ratio assuming the Osipkov–Merritt anisotropy profile for a subsample of SLACS. Additionally, the unknown 3D structures of the mass distribution and the tracer distribution are also potential sources of systematics (Cappellari 2008).

Spatially resolved velocity dispersion measurements can better constrain the anisotropy profile by breaking the mass–anisotropy degeneracy. However, spatially resolved kinematics data from IFU spectroscopy are more expensive than an integrated measurement from long-slit spectroscopy. Thus, usage of such data in lensing studies has been limited (see van de Ven et al. 2010; Barnabè et al. 2011; Shajib et al. 2023, for example of IFU data being combined with strong lensing).

### Comparison with Galaxy Simulations

The past decade has seen significant progress in understanding galaxies’ structure and formation. Within the $\Lambda$CDM paradigm, there is general agreement regarding the gravitational aspect of galaxy formation. The ‘gastrophysics’ is less well understood and requires subgrid models to simulate, but still, the galaxies formed in simulations like Illustris (Vogelsberger et al. 2014), FIRE (Hopkins et al. 2014), EAGLE (Crain et al. 2015), IllustrisTNG (Nelson et al. 2019) are much more credible than previous generations of simulated galaxies. There are also equilibrium galaxy models, including stars, gas, and dark matter, of which the AGAMA (Vasiliev 2019) simulations are arguably the most sophisticated.

Despite the great advances in the fidelity of the simulations, there have been discrepancies between the simulated predictions and the observed properties of galaxies. In particular, simulations have not been successful yet in reproducing the observed distributions of the logarithmic slope $\gamma_{\mathrm{pl}}$ and the dark matter fraction $f_{\mathrm{dm}}$ simultaneously. A no-feedback or weak feedback prescription was required in some of the simulations to reproduce the $\gamma_{\mathrm{pl}}$ distribution, which, however, led to underestimating $f_{\mathrm{dm}}$ compared to the observations due to overestimating the star formation efficiency (e.g., Naab et al. 2007; Duffy et al. 2010; Johansson et al. 2009). Similarly, matching the $f_{\mathrm{dm}}$ distribution required strong feedback prescriptions, but these produce too shallow $\gamma_{\mathrm{pl}}$ compared to the observed distribution. More recently, the IllustrisTNG simulation reproduced a $f_{\mathrm{dm}}$–$\gamma_{\mathrm{pl}}$ distribution that is consistent with the strong lensing observations if the stellar IMF corresponds to the Salpeter IMF (Wang et al. 2020; Shajib et al. 2021). In contrast, Mukherjee et al. (2022), find that the EAGLE and SLACS $f_{\mathrm{dm}}$–$\gamma_{\mathrm{pl}}$ distributions agree while using a Chabrier IMF, supporting the important role played by feedback and sub-grid physics in reproducing this relation.

**Fig. 10:**
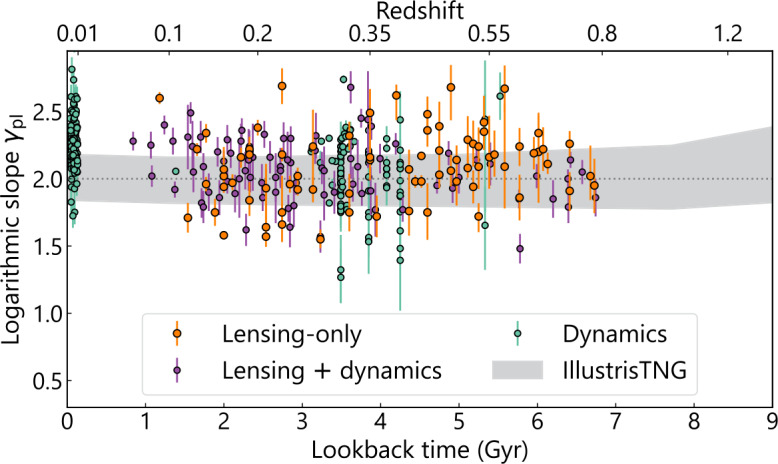
Comparison of measured logarithmic slopes $\gamma _{\mathrm{pl}}$ of the total density profile at different redshifts between lensing-only measurements (orange points), lensing–dynamics measurements (purple points), dynamical measurements (emerald points), and the IllustrisTNG simulation (grey shaded region; Wang et al. 2020). The lensing-only measurements are from the SLACS, SL2S, and BELLS samples (Tan et al. 2024). The lensing–dynamics measurements are from the SLACS and SL2S samples (Auger et al. 2010b; Sonnenfeld et al. 2013). The dynamical measurements are from the ATLAS^3D^, the Frontier Fields, and the Middle Ages Galaxy Properties with Integral field spectroscopy (MAGPI) surveys (Poci et al. 2017; Derkenne et al. 2021, 2023). The horizontal dotted line traces $\gamma _{\mathrm{pl}}=2$, the isothermal case

Furthermore, there has been an apparent tension in the redshift evolution of the logarithmic slope $\gamma_{\mathrm{pl}}$ between observations and simulations. Strong lensing observations report a steepening of $\gamma_{\mathrm{pl}}$ with decreasing redshift at $z < 1$ (Ruff et al. 2011; Sonnenfeld et al. 2013). Such a steepening would require dissipative processes through wet mergers along the evolutionary track of elliptical galaxies. Simulations instead find no evidence for redshift evolution of $\gamma_{\mathrm{pl}}$ below $z = 1$, or a slightly shallowing trend in $\gamma_{\mathrm{pl}}$ with decreasing redshift (see Fig. 10; Xu et al. 2017; Remus et al. 2017; Wang et al. 2020). Strong lensing selection effects could be a potential source of this discrepancy (Sonnenfeld et al. 2015). Moreover, this tension vanishes if the same strong lensing analysis is applied to the simulated galaxies from Illustris and Magneticum to extract $\gamma_{\mathrm{pl}}$ by combining lensing and kinematic information (Xu et al. 2017; Remus et al. 2017). Therefore, the modeling systematics in the joint analysis of lensing and dynamical observables cannot be ruled out as another potential source of the above tension. For an example of modeling systematic, if the true mass distribution in lensing elliptical galaxies is not an accurate power law, then lensed images are formed at different galactocentric radii as the lens and source redshifts vary (for a relevant test of systematic, see Gomer et al. 2022). In that case, minor deviations from the adopted power-law model may mimic an (absence of) evolution of the galaxy’s total density profile with redshift. To account for this effect, Dutton and Treu (2014) suggest using a mass-weighted slope.

An alternative approach to compare observed galaxy properties to theoretical models is the semi-empirical one adopted by Shankar et al. (2017, 2018). These authors find their semi-empirical model for massive elliptical galaxies to be consistent with un-contracted NFW halos and the Salpeter IMF, corroborating with the majority of the previous literature (see Sect. 4.1.2 and Sect. 4.2). These authors also investigate the redshift dependence of the logarithmic slope $\gamma _{\mathrm{pl}}$ mentioned above and find that a redshift dependence of the Sérsic index is necessary to explain it. In contrast, the selection function does not contribute to producing the redshift-dependent trend in $\gamma _{\mathrm{pl}}$.

The specific importance of various baryonic processes in the evolution of ellipticals is still an open question. The simulations would require further fine-tuning to consistently reproduce all of their observable properties. However, modeling systematics and the selection function must be appropriately considered for accurate comparison between simulations and observations. The first results from a statistical framework jointly considering simulations and observations to infer the galaxy evolution scenario indicate that AGN feedback is essential (Denzel et al. 2021a), but application to a large sample of lenses is required.

An additional usage of simulated galaxies is to take them as the deflector galaxies in synthesizing strong-lensing observables for testing and validating the assumptions made in the simply parametrized mass models (e.g., Enzi et al. 2020; Ding et al. 2021a). These investigations often find the simple power-law parametrization to be inadequate to accurately describe the data (e.g., He et al. 2023). However, numerical inadequacies in synthesizing the strong-lensing observables can also potentially hamper such investigations (Van de Vyvere et al. 2020). Alternatively, simply parametrized lens models can also be tested based on independent empirical observations, for example, using high-resolution imaging of nearby ellipticals (Gilman et al. 2017), or using dynamical models from highly resolved IFU spectroscopy of ellipticals (e.g., Cao et al. 2022; Poci and Smith 2022). In the future, exquisite high-resolution imaging from the *JWST* or extremely large telescopes, or advanced dynamical models, such as Schwarzschild models (extending the original method of Schwarzschild 1979), will provide powerful means to carry out these important validation tests.

## Concluding Remarks

In this review article, we have provided a review of the applications of galaxy-scale strong lensing in astrophysics and cosmology. Inevitably, some special sub-topics within the field of galaxy-scale strong lensing have evolved into proper research fields, having acquired a methodology and literature extensive enough to warrant a dedicated review. These are: detecting dark matter substructures and linking their properties to the dark matter particle, and measuring $H_{\mathrm{0}}$ and other cosmological parameters through time delays, examined in Vegetti et al. (2023) and Birrer et al. (2024), respectively.

We started with a brief historical overview in Sect. 2. Then, in Sect. 3, we have discussed both strong-lensing and complementary non-strong-lensing observables and methodologies to model and extract meaningful results from such data. The most available and informative data for galaxy-scale strong lenses come from imaging. We reviewed the most common modeling methods found in the literature to model such data and constrain galaxy properties from lensed arcs, and in some cases with the inclusion of multiple images of a point-like source. Next, we reviewed the main scientific results from the literature on the astrophysics of galaxies in Sect. 4 and on cosmology in Sect. 5. We then discussed the currently open questions and provided future outlooks in Sect. 6.

The open questions presented in Sect. 6 provide exciting opportunities for the near future. Several large-area sky surveys – namely the *Rubin*, *Euclid*, and *Roman* observatories – will discover thousands of new galaxy-scale lensing systems. These treasure troves of data will provide the necessary statistical power to shed light on the open questions on galaxy evolution and cosmology.

## Footnotes

1 https://lweb.cfa.harvard.edu/castles/.

2 There are a handful cluster-scale lenses included in this sample as well.

3 Lensed arcs from extended sources are also vulnerable to dust extinction.

4 This is also referred to as the penalty or loss function.

5 There are at least five cases of central images where the lens has two or more main lensing galaxies.
